# Complement-activating donor-specific anti-HLA antibodies and solid organ transplant survival: A systematic review and meta-analysis

**DOI:** 10.1371/journal.pmed.1002572

**Published:** 2018-05-25

**Authors:** Antoine Bouquegneau, Charlotte Loheac, Olivier Aubert, Yassine Bouatou, Denis Viglietti, Jean–Philippe Empana, Camilo Ulloa, Mohammad Hassan Murad, Christophe Legendre, Denis Glotz, Annette M. Jackson, Adriana Zeevi, Stephan Schaub, Jean–Luc Taupin, Elaine F. Reed, John J. Friedewald, Dolly B. Tyan, Caner Süsal, Ron Shapiro, E. Steve Woodle, Luis G. Hidalgo, Jacqueline O’Leary, Robert A. Montgomery, Jon Kobashigawa, Xavier Jouven, Patricia Jabre, Carmen Lefaucheur, Alexandre Loupy

**Affiliations:** 1 Paris Translational Research Center for Organ Transplantation INSERM Unit 970, Paris, France; 2 Department of Nephrology, Dialysis and Transplantation, CHU de Liège, Liège, Belgium; 3 Department of Kidney Transplantation, Necker Hospital, Paris Descartes University, and Assistance Publique–Hôpitaux de Paris (AP–HP), Paris, France; 4 Division of Nephrology, Geneva University Hospitals, Geneva, Switzerland; 5 Department of Nephrology and Kidney Transplantation, Saint–Louis Hospital, Paris Diderot University, AP–HP, Paris, France; 6 Hospital Barros Luco Trudeau, Santiago, Chile et Clinica Alemana de Santiago, Chile; 7 Mayo Clinic Evidence–based Practice Center, Mayo Clinic, Rochester, Minnesota, United States of America; 8 Immunogenetics Laboratory, Johns Hopkins University School of Medicine, Baltimore, Maryland, United States of America; 9 Department of Pathology, Surgery and Immunology at University of Pittsburgh Medical Center, Pittsburgh, Pennsylvania, United States of America; 10 Clinic for Transplantation Immunology and Nephrology, University Hospital Basel, Basel, Switzerland; 11 Department of Immunology and Histocompatibility, CHU Paris–GH St–Louis Lariboisière, Paris, France; 12 Department of Pathology and Laboratory Medicine, UCLA Immunogenetics Center, David Geffen School of Medicine, University of California, Los Angeles, California, United States of America; 13 Northwestern University Feinberg School of Medicine, Comprehensive Transplant Center, Division of Transplant Surgery, Chicago, Illinois, United states of America; 14 Division of Nephrology, Department of Medicine, Stanford University, Stanford, California, United States of America; 15 Institute of Immunology, Heidelberg University, Department of Transplantation Immunology, Heidelberg, Germany; 16 Kidney/Pancreas Transplant Program, Mount Sinai Hospital, Recanati Miller Transplantation Institute, New York, New York, United States of America; 17 Division of Transplantation, Department of Surgery, and Division of Hematology and Oncology, Department of Internal Medicine, University of Cincinnati College of Medicine, Cincinnati, Ohio, United States of America; 18 Department of Laboratory Medicine and Pathology and Alberta Transplant Applied Genomics Center, Edmonton, Alberta, Canada; 19 Annette C. & Harold C. Simmons Transplant Institute, Baylor University Medical Center, Dallas, Texas, United States of America; 20 The NYU Transplant Institute, New York University Langone Medical Center, New York, New York, United States of America; 21 Cedars–Sinai Heart Institute, Los Angeles, California, United States of America; 22 Department of Cardiology and Global Health Unit European Georges Pompidou Hospital, Paris; 23 SAMU of Paris, Necker Hospital Paris, France; 24 Paris Descartes University, Paris, France; 25 AP–HP, Paris, France; Royal Derby Hospital, UNITED KINGDOM

## Abstract

**Background:**

Anti-human leukocyte antigen donor-specific antibodies (anti-HLA DSAs) are recognized as a major barrier to patients’ access to organ transplantation and the major cause of graft failure. The capacity of circulating anti-HLA DSAs to activate complement has been suggested as a potential biomarker for optimizing graft allocation and improving the rate of successful transplantations.

**Methods and findings:**

To address the clinical relevance of complement-activating anti-HLA DSAs across all solid organ transplant patients, we performed a meta-analysis of their association with transplant outcome through a systematic review, from inception to January 31, 2018. The primary outcome was allograft loss, and the secondary outcome was allograft rejection. A comprehensive search strategy was conducted through several databases (Medline, Embase, Cochrane, and Scopus).

A total of 5,861 eligible citations were identified. A total of 37 studies were included in the meta-analysis. Studies reported on 7,936 patients, including kidney (*n* = 5,991), liver (*n* = 1,459), heart (*n* = 370), and lung recipients (*n* = 116). Solid organ transplant recipients with circulating complement-activating anti-HLA DSAs experienced an increased risk of allograft loss (pooled HR 3.09; 95% CI 2.55–3.74, *P* = 0.001; I^2^ = 29.3%), and allograft rejection (pooled HR 3.75; 95% CI: 2.05–6.87, *P* = 0.001; I^2^ = 69.8%) compared to patients without complement-activating anti-HLA DSAs. The association between circulating complement-activating anti-HLA DSAs and allograft failure was consistent across all subgroups and sensitivity analyses. Limitations of the study are the observational and retrospective design of almost all included studies, the higher proportion of kidney recipients compared to other solid organ transplant recipients, and the inclusion of fewer studies investigating allograft rejection.

**Conclusions:**

In this study, we found that circulating complement-activating anti-HLA DSAs had a significant deleterious impact on solid organ transplant survival and risk of rejection. The detection of complement-activating anti-HLA DSAs may add value at an individual patient level for noninvasive biomarker-guided risk stratification.

**Trial registration:**

National Clinical Trial protocol ID: NCT03438058.

## Abstract 2

In a systematic review and meta-analysis, Alexandre Loupy and colleagues study antibody biomarkers of solid organ transplantation outcomes.

## Author summary

**Why was this study done?:**

- Allograft rejection is a major threat to allografts, with consequences for the patients in terms of mortality and morbidity.
- Over the last decade, studies on solid organ transplant patients have reported that complement-activating anti-human leukocyte antigen donor-specific antibodies (anti-HLA DSAs) are highly associated with allograft rejection and failure, with varying magnitudes of effect.

**What did the researchers do and find?:**

- This study was designed to evaluate the clinical relevance of complement-activating anti-HLA DSAs at a population level and across the entire solid organ transplants spectrum (kidney, liver, heart, and lung transplant patients).
- The present meta-analysis, including 37 studies and 7,936 patients, provides evidence that circulating complement-activating anti-HLA DSAs are a major determinant of long-term allograft rejection and allograft failure.

**What do these findings mean?:**

- These results suggest that circulating complement-activating anti-HLA DSAs are potential noninvasive biomarkers to stratify the risk for allograft failure and rejection.
- Further research will be needed to investigate the possibility that the detection of these antibodies might have therapeutic significance and could provide opportunities for a pathogenesis-driven approach to prevention and/or treatment of rejection for solid organ transplant recipients.

## Introduction

Organ transplantation is the treatment of choice for many patients with end-stage chronic disease, which is an increasing burden on industrialized and newly industrialized countries [1,2]. Despite substantial progress in the development of effective immunosuppressive regimens, thousands of allografts fail every year worldwide due to rejection, with immediate consequences in terms of mortality, morbidity, and billions in extra costs to healthcare systems [3,4]. In the past decade, the role of circulating anti-human leukocyte antigen donor-specific antibodies (anti-HLA DSAs) has been increasingly recognized as a major contributing factor to allograft rejection [5] and long-term allograft failure [6–9] in kidney transplantation [10], with the same important associations more recently appreciated in lung [11], heart [7–12], liver [13], intestinal [14], and pancreas transplants [15].

However, not all antibodies are equal in terms of pathogenicity, and they exert a heterogeneous influence on organ allograft outcomes, ranging from acute forms of rejection leading to immediate allograft dysfunction and early allograft loss to more indolent or subclinical forms leading to progressive allograft deterioration.

The inconsistent effects of anti-HLA antibodies on allograft outcomes, which limit their prognostic value, has recently led to attempts to refine their assessment on the basis of pathogenic characteristics to determine which anti-HLA DSAs carry the highest risk for adverse transplant outcomes. Among the notable characteristics of HLA antibodies, their capacity to activate complement has been suggested as a potential factor directing their pathogenicity in the rejection process [16]. Data support that circulating anti-HLA DSAs have the ability to activate complement by their complement component 1q (C1q), C3d, and C4d complement fraction-binding capacities or by their immunoglobulin G3 (IgG3) subclass component, which are associated with an increased risk of antibody-mediated rejection (ABMR) and allograft loss in solid organ transplant recipients [16–25]. However, prior studies have reported different magnitudes of effect for these antibodies, ranging from strong effects to the absence of associations with allograft outcomes [18,19,26–30], limiting their implementation in clinical practice. Greater precision in predicting allograft outcomes using a mechanistically informed, noninvasive biomarker generalizable to diverse solid organ transplants has been identified as a major goal by professional societies (e.g., the European Society of Organ Transplantation, the American Society for Transplantation, and the American Society of Transplant Surgeons), agencies (e.g., the European Medicine Agency and the Food and Drug Administration) [31], and consortia [32]. These groups have pointed to the need for such biomarkers as vital both to optimizing allocation policy and to better stratifying the risk of long-term allograft failure for individual patients. This meta-analysis aims to evaluate the role of complement-activating anti-HLA DSAs on graft survival and graft rejection across the entire spectrum of solid organ transplants.

## Methods

This meta-analysis is reported in adherence with the Preferred Reporting Items for Systematic Reviews and Meta-Analyses (PRISMA) and the reporting Meta-Analyses of Observational Studies in Epidemiology (MOOSE) [33,34].

### Data sources and searches

A comprehensive search was designed and conducted by an experienced librarian with input from the study investigators. The complete protocol of the research strategy was prespecified and the analysis plan prospectively written (S1 Text). Controlled vocabulary supplemented with keywords was used to search for complement-activating anti-HLA DSAs in human solid organ transplantation in any language. The following databases were included: Ovid MEDLINE In-Process & Other Non-Indexed Citations, Ovid MEDLINE, Ovid EMBASE, Ovid Cochrane Central Register of Controlled Trials, Ovid Cochrane Database of Systematic Reviews, and Scopus. The research was conducted from database inception to January 31, 2018. Complement-activating anti-HLA DSAs were defined by their capacity to activate complement cascade at different levels—C1q [23], C3d [35], C4d [26], or presence of IgG3 subtype [36].

The following keywords were used for the research: “solid organ transplantation,” “kidney transplantation,” “liver transplantation,” “lung transplantation,” “heart transplantation,” “intestines transplantation,” “donor specific anti-HLA antibodies,” “solid-phase assay,” “complement-activating DSA,” “C1q,” “C3d,” “C4d,” “IgG3 subclass,” “outcome,” “graft loss,” “graft survival,” “ABMR,” and “rejection.” For comprehensiveness, we also reviewed all references listed in the full-text publications and reviews on the subject that were not identified by our search criteria. An example of the research strategy in the Ovid database is described in S2 Text.

### Study selection

Studies of any relevant design and in any language on the impact of complement-activating anti-HLA DSAs on long-term graft survival and/or the risk of rejection were initially selected. The eligible studies included all solid organ transplant patients (kidney, liver, lung, heart, and intestinal transplantation), both adult or pediatric patients. Anti-HLA DSAs detected by the Luminex single-antigen bead (SAB) technique were required for the DSA detection technique. Complement-activating anti-HLA DSAs were defined according to their ability to bind C1q, C3d, C4d or their IgG3 subclass. The endpoints of interest for inclusion were either allograft loss for the primary endpoint and/or biopsy-proven rejection as a secondary endpoint. Allograft rejection was labelled either antibody-mediated or mixed-rejection as defined by the Banff international classification for kidney and liver transplants [37,38] or the International Society for Heart and Lung Transplantation (ISHLT) classification for heart and lung transplants [39]. Data on graft loss (hazard ratio [HR]) and/or allograft rejection (HR or odds ratio [OR]) were extracted when available and defined as effect sizes with their 95% confidence intervals (CIs).

The corresponding author of each eligible study was contacted and asked to provide HRs and/or ORs when these were not available in the manuscript. All initial communications with authors were based on a template explaining the study and the data required. Two separate reminders were sent unless we received a definitive response. When no answer was obtained, the study was excluded from the analysis.

We excluded unrelated articles, including those without information on complement-activating anti-HLA DSAs, duplicates, those with nonhuman results or non–solid-organ transplant data, case reports, abstract-only articles, and reviews.

Two reviewers (C Loheac and A Bouquegneau) independently assessed the potential eligibility of each of the titles and abstracts that resulted from the search and then reviewed the full texts of all potentially eligible studies. Chance-adjusted inter-reviewer agreement (kappa statistic) was calculated. All disagreements were resolved by consensus between reviewers and principal investigators (C Lefaucheur and A Loupy).

### Data extraction and quality assessment

The collected data included author name, year of publication, study size, mean or median follow-up time, mean age of population, type of complement-activating anti-HLA DSA, comparison used (patients with complement-activating anti-HLA DSAs were either compared to patients without complement-activating anti-HLA DSAs, patients with non-complement activating anti-HLA DSAs detected, or a mixed group of patients without anti-HLA DSAs and with non-complement activating anti-HLA DSAs), effect sizes (HR and/or OR) and their 95% CIs, potential confounding factors, and unadjusted and adjusted estimated risks of graft loss or graft rejection. Adjusted HRs and ORs were used when available; otherwise, univariate effect sizes were used.

We used the Newcastle–Ottawa Scale (NOS) to assess the methodological quality (i.e., risk of bias) of nonrandomized studies [40]. NOS score was calculated on the basis of the following 3 major components: the selection of the study groups and ascertainment of exposure (0 to 4 points), quality of the adjustment for confounding variables (0 to 2 points), and ascertainment of outcomes (0 to 3 points). A high NOS score represents high methodological quality. The only randomized controlled trial was assessed using the Cochrane Risk of Bias tool. Details regarding the NOS scoring system are provided in S3 Text.

### Data synthesis and analysis

Meta-analysis was performed using a random-effects model [41] because of the anticipated heterogeneity across studies. In a random-effects meta-analysis model, the effect sizes from the studies that actually were performed are assumed to represent a random sample from a particular distribution of these effect sizes and take into account both within-study variability (expressed by the CI in each study’s effect sizes) and between-study variability (heterogeneity).

The index group for comparison was patients with complement-activating anti-HLA DSAs, and they were either compared to patients with non–complement-activating anti-HLA DSAs, patients without anti-HLA DSAs detected, or a mixed group of patients without anti-HLA DSAs and with non-complement activating anti-HLA DSAs.

### Statistical heterogeneity and publication bias

Statistical heterogeneity across the studies was tested with the I^2^ index [42]. The I^2^ index describes the percentage of total variation across studies due to heterogeneity rather than chance. A value of 0% indicates no observed heterogeneity; values exceeding 50% may elicit considerable caution and warrant further analysis through subgroup analyses [43]. A low *P* value of the I^2^ test (below 0.05) provides evidence of heterogeneity of intervention effects (variation in effect estimates beyond chance). Publication bias was visually assessed using funnel plots and statistically assessed by the Egger’s bias coefficient, which weighted the regression of the intervention effect on its standard error (SE), with weights inversely proportional to the variance of the intervention effect [44]. *P* < 0.05 (2-sided) was considered statistically significant for the presence of a publication bias.

We investigated the extent to which statistical heterogeneity between results of multiple studies can be related to one or more characteristics of the studies by using metaregression [45]. Metaregression merges meta-analytic techniques with linear regression principles (predicting treatment effects using covariates). Metaregression could also explore possible causes of heterogeneity and ascertain stability of results between subgroup analyses. In the present study, we decided to adjust effect sizes on the following covariates if available: date of publication, mean fluorescence intensity (MFI) for anti-HLA DSAs, number of HLA mismatches, period of inclusion, and mean recipient age. We used the overall model *P* value to assess whether there is evidence for an association of any of the covariates with the outcome [46].

### Subgroup and sensitivity analyses

These analyses were performed to explore potential sources of heterogeneity regarding the primary outcome and to assess the consistency of our results, and the choice of the different subgroup analyses was prespecified prior to any analysis. The following subgroup analyses were considered.

#### Comparator group used

Considering the index group (complement-activating anti-HLA DSA), we analyzed separately the studies comparing patients with non–complement-activating anti-HLA DSAs or control consisting of a mixed group of patients with non–complement-activating anti-HLA DSAs and without anti-HLA DSAs.

#### Studies that used multivariable models

Studies using multivariable models for addressing the independent associations of complement activation with allograft failure were analyzed separately.

#### High versus low methodological quality studies

Articles with NOS scores ≥6 (versus lower scores) were selected as high-quality studies [47] and analyzed separately.

#### Type of organ transplanted

Kidney allograft versus all other types of transplanted organs (heart, lung, and liver allografts). We decided to gather together the groups of liver, lung, and heart transplantation because of their low number. Indeed, with a low number of studies (3 or fewer), the risk of increasing the heterogeneity is important.

#### Timing of antibody detection

Preexisting anti-HLA DSAs (defined as antibodies present before or at the time of transplantation), de novo anti-HLA DSAs (defined as antibodies present only after transplantation), or a combined group of preexisting and de novo DSAs.

#### Type of assay used for characterizing the complement-activating capacity of antibodies

Assays were characterized as anti-HLA DSA IgG subclass, C1q-binding anti-HLA DSAs, C4d-binding anti-HLA DSAs, or C3d-binding anti-HLA DSAs. Because IgG subclass and complement-binding tests may not provide the exact same information and biological properties, we performed a post hoc supplemental analysis on the impact of complement-binding anti-HLA DSAs (C1q, C3d, and C4d) and the IgG3 subclass studies and their respective associations with allograft outcome.

#### Center effect

This subgroup analysis excluded the largest cohorts (in terms of the number of patients included) [16,48,49]. We performed this analysis because larger studies could be a main driving factor for the associations found in primary analyses and could also modify overall heterogeneity.

Analyses were conducted using STATA (version 14.1; StataCorp, College Station, TX).

## Results

### Study identification and characteristics

**Fig 1:**
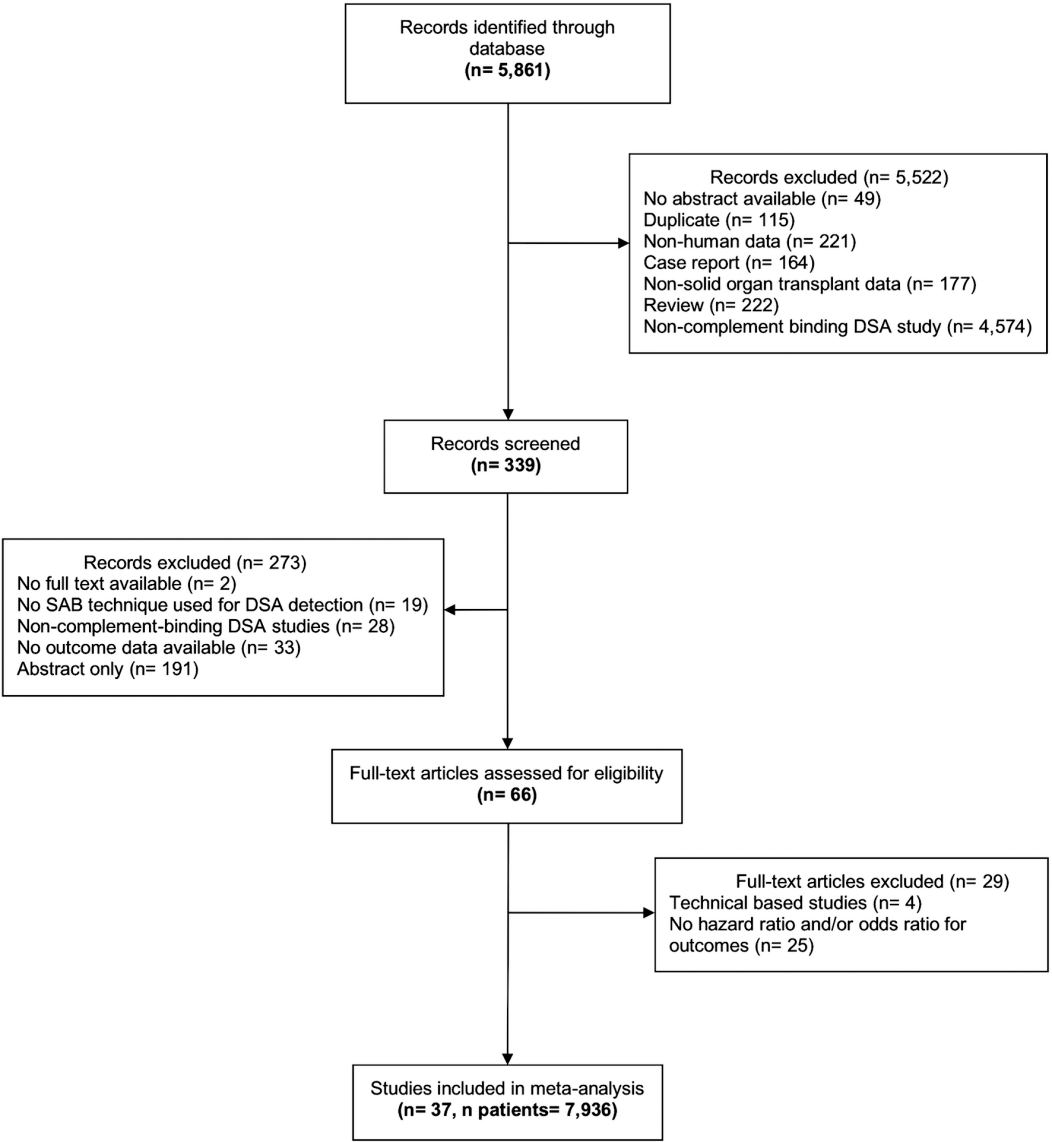
Flow chart summarizing the research strategy for study identification and selection. DSA, donor-specific antibody; SAB, single-antigen bead.

The electronic search identified 5,861 potentially relevant citations. A schematic diagram of the literature search procedure used in the present study is shown in Fig 1. The kappa statistic for study eligibility was 0.9941 between the two reviewers (SE = 0.0949). Finally, 37 studies and 7,936 patients were included in the final meta-analysis, including 24 studies with data on allograft loss, 8 studies with data on rejection, and 5 studies with both primary- and secondary-outcome data. Table 1 summarizes characteristics of the included studies. S1 Table provides a detailed characteristic of included studies.

**Table 1:** Characteristics of the 37 included studies.

First author (date of publication)	Population	Study type	Period of inclusion	Number of patients	Effect size (95% CI)
**Wahrmann et al. (2009)** [26]	Retrospective, single-center analysis of consecutive adult renal transplants selected based on the presence of pretransplant DSAs	Cohort	2001–2002	338	2.40 (0.90–6.00) for graft loss 10.10 (3.20–31.00) for rejection
**Hönger et al. (2010)** [28]	Retrospective, single-center analysis of consecutive adult renal transplant recipients with low levels of pretransplant DSAs	Cohort	1999–2004	64	0.93 (0.25–3.44) for rejection
**Sutherland et al. (2011)** [50]	Retrospective, single-center analysis of pediatric renal transplant recipients without DSAs at the time of transplantation	Cohort	2000–2008	35	5.80 (1.40–22.90) for graft loss
**Hönger et al. (2011)** [51]	Retrospective, single-center analysis of adult renal transplant recipients with high levels of DSAs pre transplant; recipients who developed ABMR within 6 months	Cohort	1999–2008	71	0.43 (0.17–1.12) for rejection
**Smith et al. (2011)** [7]	Retrospective, single-center analysis of living heart transplant recipients after 1 year of transplantation without DSAs pre transplant	Cohort	1995–2004	243	3.02 (1.11–8.23) for graft loss
**Kaneku et al. (2012)** [52]	Retrospective (2-center) analysis of adult liver transplant recipients with liver biopsies showing chronic rejection and DSA analysis at the same time	Case- control	NC	39	3.35 (1.39–8.05) for graft loss
**Bartel et al. (2013)** [53]	Retrospective, single-center analysis of 68 desensitized renal recipients who had been subjected to peritransplant desensitization	Cohort	1999–2008	68	10.10 (1.60–64.20) for rejection
**Lawrence et al. (2013)** [54]	Retrospective, single-center study of consecutive renal transplant recipients	Cohort	2005–2010	52	8.90 (1.20–65.86) for rejection
**Crespo et al. (2013)** [55]	Retrospective (2-center) analysis of renal transplant patients with pretransplant DSAs	Cohort	2006–2011	355	0.83 (0.17–4.14) for graft loss 1.44 (0.23–9.11) for rejection
**Loupy et al. (2013)** [16]	Consecutive adult patients in a retrospective (2-center) analysis; unselected global population with DSA detection before or after renal transplantation	Cohort	2004–2010	1,016	4.78 (2.69–8.49) for graft loss
**Freitas et al. (2013)** [56]	Retrospective, single-center analysis of renal transplant recipients selected on the basis of DSA detection during follow-up	Cohort	1999–2012	203	3.50 (1.30–9.50) for graft loss
**Arnold et al. (2014)** [57]	Retrospective, single-center analysis of renal transplant recipients without DSAs pre transplant and screened for de novo DSAs	Cohort	1997–2007	274	4.81 (1.65–14.03) for graft loss
**Smith et al. (2014)** [25]	Retrospective, single-center analysis of lung transplant recipients with pretransplant DSA detection	Cohort	1991–2003	63	6.43 (2.96–13.97) for graft loss
**Everly et al. (2014)** [58]	Retrospective, single-center analysis of primary renal transplant recipients without pretransplant DSA detection	Cohort	1999–2006	179	2.48 (1.02–6.04) for graft loss
**O’Leary et al. (2015)** [24]	Retrospective, single-center analysis of consecutive patients with 1-year survival post liver transplantation; one group analyzed pretransplant DSA effects, and another group analyzed the impact of de novo DSAs	Cohort	2000–2009	1,270	1.90 (1.62–3.45) for C1q for graft loss 2.40 (1.82–5.75) for IgG3 for graft loss
**Wozniak et al. (2015)** [59]	Retrospective, single-center analysis of pediatric liver transplant patients who were either nontolerant, tolerant, or stable	Cohort	NC	50	4.30 (1.10–16.40) for rejection
**Khovanova et al. (2015)** [60]	Retrospective, single-center analysis of HLA-incompatible desensitized renal transplant patients	Cohort	2003–2012	80	1.69 (0.41–6.93) for preexisting DSAs for graft loss 2.09 (0.30–14.60) for preexisting and de novo DSAs for graft loss
**Sicard et al. (2015)** [17]	Retrospective analysis of consecutive (2-center) adult renal transplant patients who developed ABMR	Cohort	2004–2012	69	2.80 (1.12–6.95) for C3d for graft loss 1.98 (0.95–4.14) for C1q for graft loss
**Thammanichanond et al. (2016)** [61]	Retrospective, single-center cohort study of patients with pre–renal transplant DSAs	Cohort	2009–2013	48	2.20 (0.61–7.85) for rejection
**Comoli et al. (2016)** [20]	Retrospective analysis of consecutive pediatric recipients; single center; first kidney transplant without any HLA antibodies in sera or at the time of transplantation	Cohort	2002–2013	114	6.91 (2.78–17.18) for rejection and C3d 13.54 (4.95–36.99) for rejection and C1q 27.80 (5.61–137.72) for graft loss and C3d 11.09 (2.25–54.64) for graft loss and C1q
**Yamamoto et al. (2016)** [62]	Retrospective analysis of renal transplant patients with de novo DSAs and surveillance biopsies	Cohort	2009–2013	43	2.60 (0.12–53.90) for rejection
**Calp–Inal et al. (2016)** [18]	Retrospective analysis; single center; consecutive renal transplant patients: Group 1 without pretransplant DSAs and Group 2 with a mix of preexisting and de novo DSAs	Cohort	2009–2012	284	4.30 (1.10–16.50) for graft loss
**Malheiro et al. (2016)** [63]	Retrospective, single-center analysis of kidney transplant patients with DSAs pre transplant	Cohort	2007–2012	60	16.80 (3.18–88.85) for rejection
**Visentin et al. (2016)** [64]	Retrospective, single-center analysis of lung transplant patients with biopsy (with demonstration of rejection) and serum available	Cohort	1999–2014	53	1.65 (0.68–3.97) for graft loss
**Kauke et al. (2016)** [30]	Retrospective, single-center analysis of patients selected based on renal biopsy-proven rejection during graft dysfunction or viremia with polyomavirus BK	Cohort	2005–2011	611	3.77 (1.40–10.16) for graft loss 4.52 (1.89–10.37) for rejection
**Bamoulid et al. (2016)** [65]	Retrospective, single-center analysis of renal transplant consecutive patients without DSAs pre transplant	Cohort	2007–2014	59	2.27 (1.05–4.91) for rejection 6.78 (0.86–53.50) for graft loss
**Fichtner et al. (2016)** [21]	Retrospective, single-center analysis of prospectively screened renal transplant pediatric patients, non-presensitized	Cohort	1999–2010	62	6.35 (1.33–30.40) for graft loss
**Guidicelli et al. (2016)** [19]	Retrospective, single-center analysis of consecutive nonsensitized kidney transplant patients	Cohort	1998–2005	346	2.99 (0.94–10.27) for graft loss
**Lefaucheur et al. (2016)** [48]	Retrospective analysis of consecutive patients (2-center); renal transplant patients were unselected	Cohort	2008–2010	125	4.80 (1.70–13.30) for IgG3 for graft loss 3.60 (1.10–11.70) for C1q for graft loss
**Viglietti et al. (2017)** [49]	Retrospective analysis of consecutive patients (2-center); renal transplant recipients were unselected	Cohort	2008–2011	851	4.25 (1.88–9.61) for IgG3 for graft loss 3.60 (1.71–7.59) for C1q for graft loss
**Wiebe et al. (2017)** [27]	Retrospective analysis of consecutive adult and pediatric renal transplant patients, single center; patients without pretransplant sensitization	Cohort	1999–2012	70	1.06 (0.50–2.40) for graft loss
**Moktefi et al. (2017)** [66]	Retrospective analysis (2-center) of patients selected based on the development of acute renal ABMR and the presence of DSAs	Cohort	2005–2012	48	0.79 (0.25–2.44) for graft loss
**Sicard et al. (2017)** [67]	Retrospective analysis of consecutive adult renal transplant patients (2-center) with unselected patients	Cohort	2004–2012	52	3.71 (1.27–10.80) for graft loss
**Das et al. (2017)** [68]	Retrospective, single-center analysis of pediatric heart transplant without DSAs pre transplantation and at the time of transplantation	Cohort	2005–2014	127	3.20 (1.34–7.86) for graft loss
**Couchonnal et al. (2017)** [69]	Retrospective analysis; single-center analysis of consecutive pediatric liver transplant selected on the presence of DSAs during follow-up	Cohort	1990–2014	100	4.12 (0.95–17.89) for graft loss
**Bailly et al. (2017)** [70]	Retrospective analysis of multicenter, prospective, randomized, double-blind, placebo-controlled, parallel-group trials; patients selected on the basis of renal ABMR development and DSA detection; patients treated either with standard of care (PP plus IVIg) or rituximab plus standard of care	Cohort	2008–2011	25	3.70 (0.80–17.00) for graft loss
**Molina et al. (2017)** [71]	Retrospective analysis; single-center analysis of consecutive adult kidney transplant patients selected on pretransplant DSA detection	Cohort	1995–2009	389	4.01 (2.33–6.92) for graft loss

Effect sizes refer to HR for graft survival and OR for rejection appearance.

**Abbreviations:** ABMR, antibody-mediated rejection; C1q, complement component 1q; CI, confidence interval; DSA, donor-specific antibody; HLA, human leukocyte antigen; HR, hazard ratio; IgG3, immunoglobulin G3; IVIg: intravenous immunoglobulin; NC, not communicated; OR, odds ratio; PP, plasmapheresis.

Overall, 22 (59.5%) studies originated from Europe, 9 (24.3%) originated from North America, 4 (10.8%) originated from the United Kingdom, and 2 (5.4%) originated from Asia. The patients included were kidney recipients (*n* = 5,991; 75.5%), liver recipients (*n* = 1,459; 18.4%), heart recipients (*n* = 370; 4.7%), and lung recipients (*n* = 116; 1.4%). None of the studies included patients with intestine or pancreas transplantation. Complement-activating anti-HLA DSAs were assessed by their capacity to bind C1q (19 studies), C4d (6 studies), or C3d (4 studies) or by their IgG subclass composition (8 studies). Six studies simultaneously analyzed 2 complement-activating anti-HLA DSA assays [17,20,24,48,49,56]. The mean patient follow-up time post transplantation was 71.2 ± 32.3 months. None of the studies included were sponsored or conducted by diagnostic companies involved in the manufacture or sale of complement-activating antibody assays. Nineteen authors were contacted and asked for supplementary data, and 63% of them provided with the requested information.

**Fig 2:**
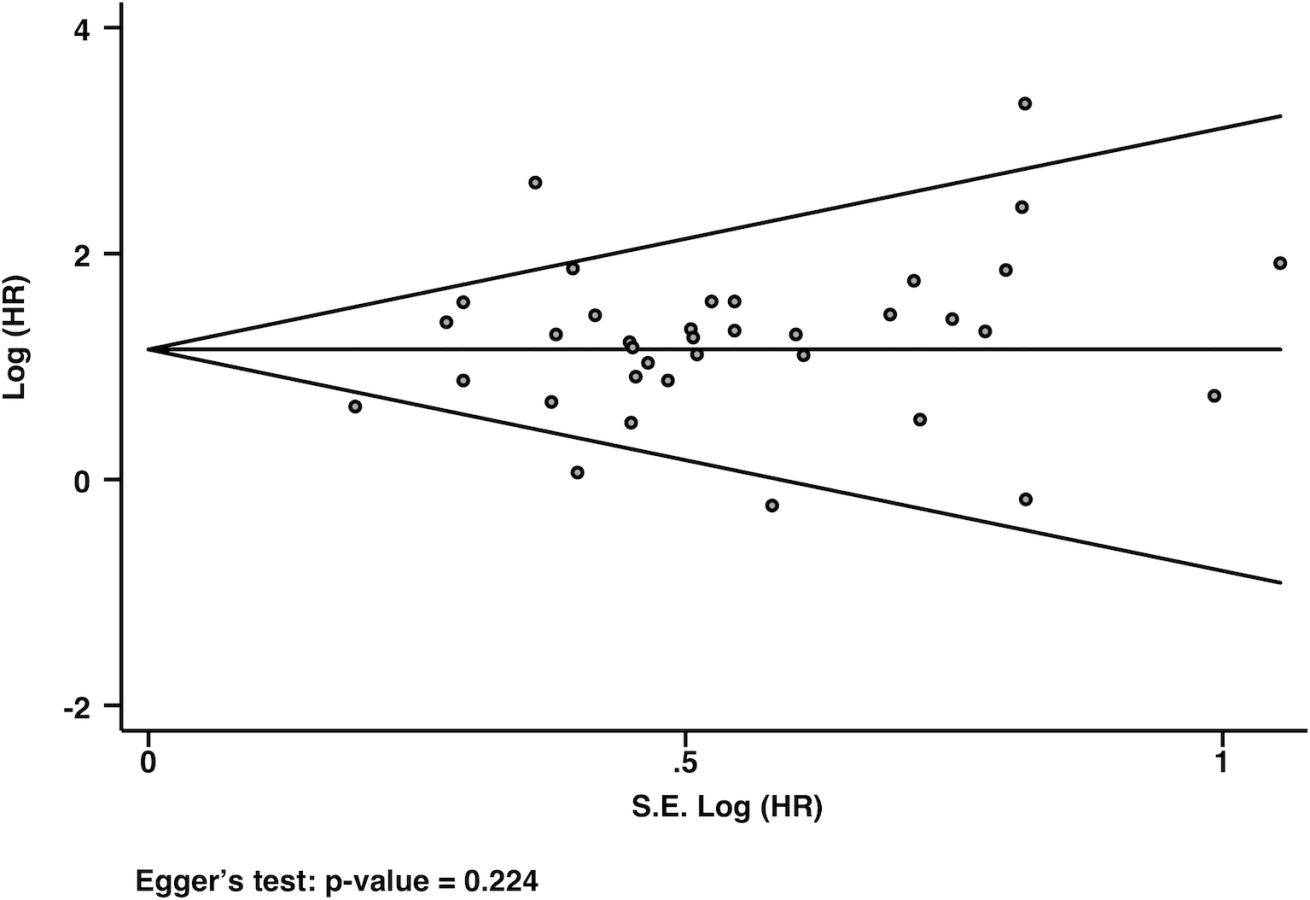
Funnel plot representing the analysis for publication bias with Egger’s test for bias. Each dot represents a study; the y-axis represents study precision (95% CIs), and the x-axis shows the SE of the HR. CI, confidence interval; HR, hazard ratio; SE: standard error.

The funnel plot presented in Fig 2 demonstrates the absence of a publication bias (Egger’s test *P* = 0.224). The randomized controlled trial was of moderate quality [70]. The NOS scores for quality assessments of the included studies are presented in S2 Table. The median NOS score was 6 (minimum 3, maximum 9), with 2.8%, 2.8%, 19.4%, 38.9%, 22.2%, 11.1%, and 2.8% of studies having a NOS score of 3, 4, 5, 6, 7, 8, and 9, respectively.

### Complement-activating anti-HLA DSA status and outcomes

#### Risk of allograft loss according to complement-activating anti-HLA DSA status

**Fig 3:**
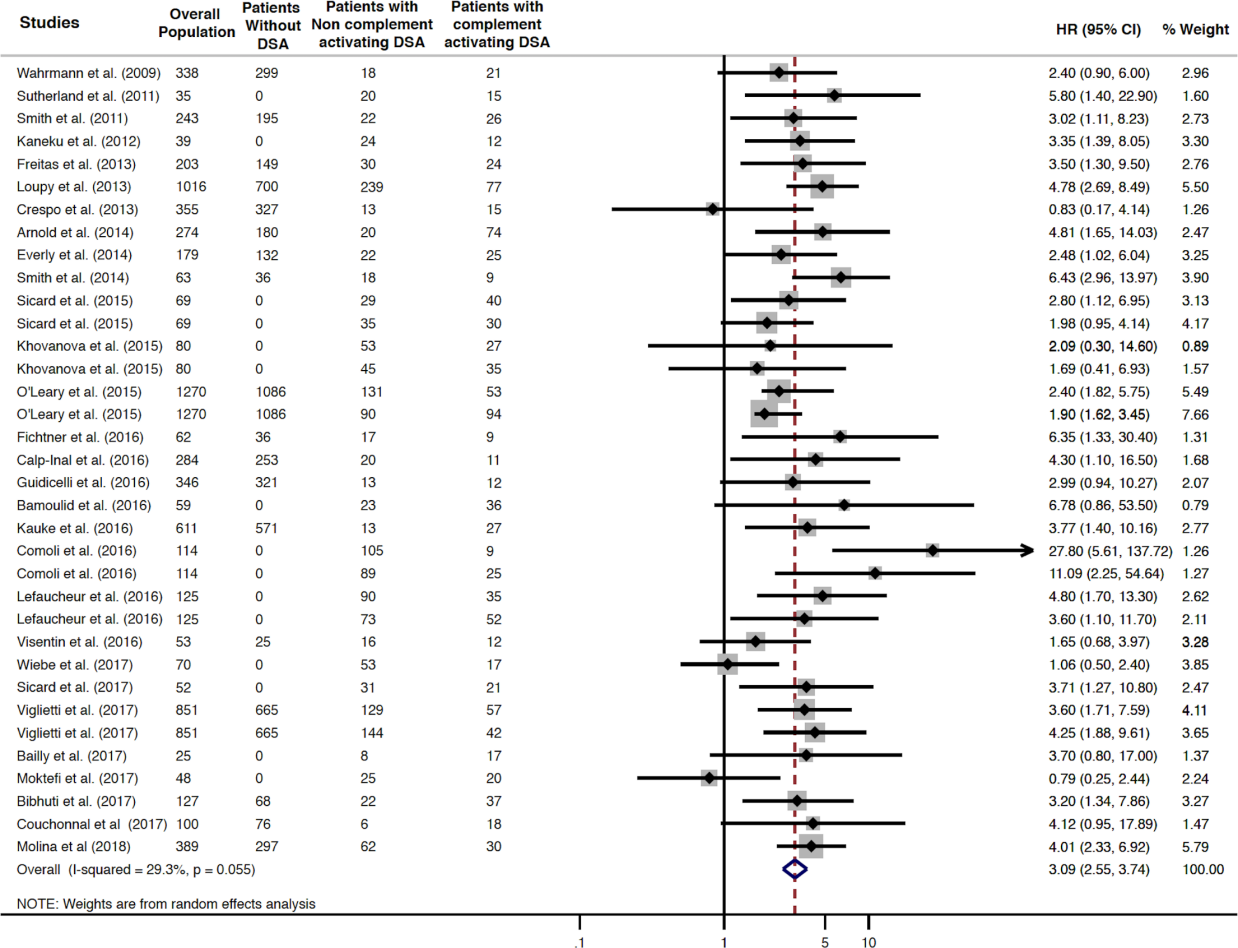
Association between circulating complement-activating anti-HLA DSAs and the risk of allograft loss. Fig 3 shows the forest plot of the association between complement-activating anti-HLA DSAs and the risk of allograft loss for each study and overall (*n* = 29). Studies are listed by date of publication. Number of patients are listed in the 3 cohort columns. The black diamond-shaped boxes represent the HR for each individual study. The grey boxes around the black diamond represent the weight of the study, and lines represent the 95% CI for individual studies. The blue diamond at the end represents the pooled HR. The number of patients in the overall population does not correspond to the sum of the different groups for the studies of Kaneku et al. (2012) (3 patients), Sicard et al. (2015) (4 patients), and Moktefi et al. (2017) (3 patients) either because the data for these patients were missing or because they were not involved in the analysis. CI, confidence interval; DSA, donor-specific antibody; HLA, human leukocyte antigen; HR, hazard ratio.

Patients with complement-activating anti-HLA DSAs had a 3.09-fold increased risk of long-term allograft loss compared to patients without anti-HLA DSAs, patients with non–complement-activating anti-HLA DSAs, and a mixed group including patients without anti-HLA DSAs and with non–complement-activating anti-HLA DSAs (HR 3.09; 95% CI 2.55–3.74, *P* = 0.001; I^2^ = 29.3%) (Fig 3).

#### Risk of allograft rejection according to complement-activating anti-HLA status

**Fig 4:**
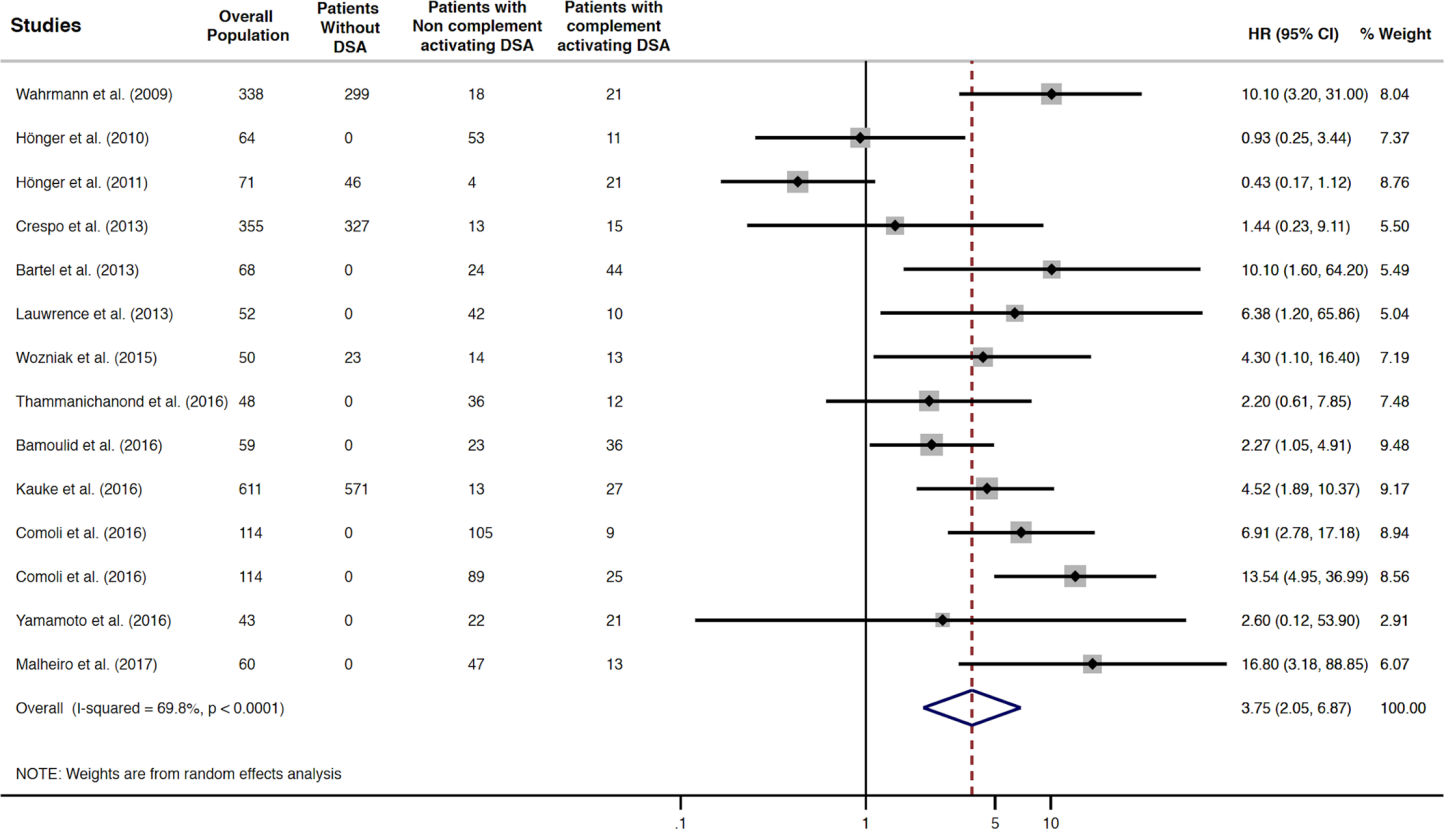
Association between complement-activating anti-HLA DSAs and the risk of rejection. Fig 4 shows the forest plot of the association between complement-activating anti-HLA DSAs and the risk of rejection for each study and overall (*n* = 13). Studies are listed by date of publication. The black diamond-shaped boxes represent the HR for each individual study. The grey boxes around the black diamond represent the weight of the study, and lines represent the 95% CI for individual studies. The blue diamond at the end represents the overall HR. CI, confidence interval; DSA, donor-specific antibody; HLA, human leukocyte antigen; HR, hazard ratio.

Patients with complement-activating anti-HLA DSAs had a 3.75-fold increased risk of allograft rejection compared to patients without anti-HLA DSAs, patients with non–complement-activating anti-HLA DSAs, and a mixed group including patients without anti-HLA DSAs and with non–complement-activating anti-HLA DSAs (HR 3.75; 95% CI 2.05–6.87, *P* = 0.001; I^2^ = 69.8%) (Fig 4).

### Subgroup and sensitivity analyses

Subgroup and sensitivity analyses were performed on the outcome of graft loss to confirm the consistency of the results and explain some of the heterogeneity found in the overall results. Table 2 summarizes the different effect sizes for the different subgroup analyses.

**Table 2:** Effect sizes related to the different subgroup analyses.

Subgroup analyses for allograft survival		Effect size	95% CI	I^2^ *P* value
**Effect of complement-activating anti-HLA DSAs in studies with high or low methodological quality**	High–methodological quality studies NOS ≥ 6	2.87	2.42–3.39	3.1% *P* = 0.418
	Low–methodological quality studies NOS < 6	3.82	1.75–8.33	67.8% *P* = 0.005
**Effect of complement-activating anti-HLA DSAs in studies with different comparators used**	Studies comparing index group and patients with non–complement-activating anti-HLA DSAs	2.94	2.04–4.23	41.1% *P* = 0.036
	Studies comparing index group and patients with non–complement-activating anti-HLA DSAs and without anti-HLA DSAs	3.60	2.74–4.73	0.0% *P* = 0.462
**Effect of complement-activating anti-HLA DSAs according to the type of solid organ transplant**	Kidney transplantation studies only	3.26	2.58–4.11	26.6% *P* = 0.102
	Heart, lung, and liver transplantation studies	2.71	1.98–3.72	29.3% *P* = 0.194
**Effect of complement-activating anti-HLA DSAs according to the timing of antibody detection**	Preexisting DSAs	2.67	1.79–4.00	52.7% *P* = 0.048
	Preexisting and de novo DSAs	3.18	2.49–4.05	0.0% *P* = 0.458
	De novo DSAs	3.65	2.45–5.44	38.0% *P* = 0.081
**Effect of complement-activating anti-HLA DSAs according to the type of test used for detecting complement-activating antibodies**	C1q	2.80	2.11–3.71	42.1% *P* = 0.028
	C4d	3.82	2.05–7.11	29.8% *P* = 0.240
	C3d	5.04	2.10–12.07	51.2% *P* = 0.105
	IgG3	3.11	2.29–4.22	0.0% *P* = 0.868
**Center effect**		2.90	2.33–3.60	31.8% *P* = 0.050

Table 2 summarizes the effect sizes observed in the different subgroup analyses described in the Materials and methods. Effect sizes refer to HR for graft survival and OR for rejection appearance. Index group refers to patients with complement-activating anti-HLA DSAs.

**Abbreviations:** C1q, complement component 1q; CI, confidence interval; DSA, donor-specific antibody; HLA, human leukocyte antigen; HR, hazard ratio; I^2^, heterogeneity; IgG3, immunoglobulin G3; NOS, Newcastle–Ottawa scale; OR, odds ratio.

#### Effect of the complement-activating anti-HLA DSAs in studies with different comparators used

Sensitivity analysis restricted to studies with different comparators used demonstrated consistent results regarding the association between complement-activating anti-HLA DSAs and risk of allograft loss, with a pooled HR of 2.94 for patients with complement-activating anti-HLA DSAs compared to patients with non–complement-activating anti-HLA DSAs (95% CI 2.04–4.23, *P* = 0.001; I^2^ = 41.1%) (S1 Fig). The pooled HR for patients with complement-activating anti-HLA DSAs compared to patients with a mixed group of patients without DSAs and with non–complement-activating DSAs was 3.60 (95% CI 2.74–4.73, *P* = 0.001; I^2^ = 0.0%) (S2 Fig).

Regarding the risk of rejection, the pooled HR for patients with complement-activating anti-HLA DSAs compared to patients with non–complement-activating anti-HLA DSAs was 4.24 (95% CI 2.23–8.06, *P* = 0.001; I^2^ = 55.0%) (S3 Fig).

#### Multivariable models: Independent prognostic value of complement-activating anti-HLA DSA

**Fig 5:**
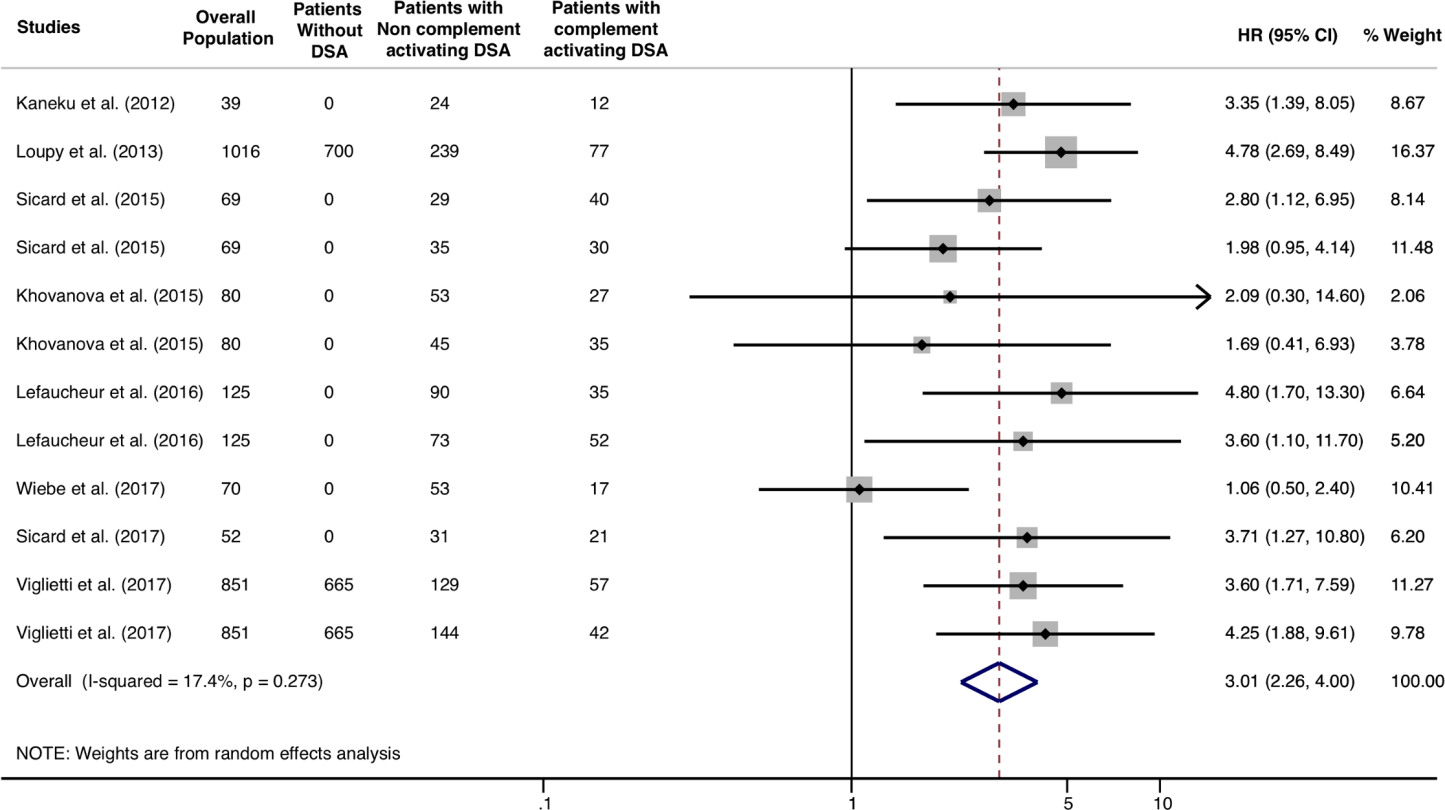
Association of circulating complement-activating anti-HLA DSAs with the risk of allograft loss in selected studies with multivariable models including MFI and complement-activating anti-HLA DSA. Fig 5 shows the forest plot of the association between complement-activating anti-HLA DSAs and the risk of allograft loss in studies with multivariable models including MFI and complement-activating anti-HLA DSA (*n* = 8). Studies are listed by date of publication. The black diamond-shaped boxes represent the HR for each individual study. The grey boxes around the black diamond represent the weight of the study, and lines represent the 95% CI for individual studies. The blue diamond at the end represents the overall HR. The number of patients in the overall population does not correspond to the sum in the different groups for the studies of Kaneku et al. (2012) (3 patients) and Sicard et al. (2015) (4 patients) either because the data for these patients were missing or because they were not involved in the analysis. CI, confidence interval; DSA, donor-specific antibody; HLA, human leukocyte antigen; HR, hazard ratio; MFI, mean fluorescence intensity.

When selecting studies that performed multivariable models, adjusting complement-activating anti-HLA DSA status on pan-IgG anti-HLA DSA level defined by the MFI, the presence of complement-activating anti-HLA DSAs remained significantly and independently associated with an increased risk of allograft loss (HR 3.01; 95% CI 2.26–4.0, *P* = 0.001), and the heterogeneity across studies decreased from 29.3% to 17.4% (Fig 5).

#### Effect of complement-activating anti-HLA DSAs in studies with high methodological quality

Sensitivity analysis restricted to studies with high methodological quality (NOS score ≥6) demonstrated consistent results regarding the association between complement-activating anti-HLA DSAs and the risk of long-term allograft loss, with a pooled HR of 2.87 (95% CI 2.42–3.39, *P* = 0.001; I^2^ = 3.1%) (S4 Fig). Sensitivity analysis restricted to studies with lower methodological quality (NOS score ≤5) demonstrated that complement-activating anti-HLA DSAs were associated with the risk of long-term allograft loss, with a pooled HR of 3.82 (95% CI 1.75–8.33, *P* = 0.001; I^2^ = 67.8%).

#### Effect of complement-activating anti-HLA DSAs according to the type of solid organ transplant

Sensitivity analysis restricted to kidney allograft recipients demonstrated an increased risk of allograft loss associated with the presence of complement-activating anti-HLA DSAs, with a pooled HR of 3.26 (95% CI 2.58–4.11, *P* = 0.001; I^2^ = 26.6%) (S5 Fig). The analysis including heart, lung, and liver recipients showed a pooled HR of 2.71 (95% CI 1.98–3.72, *P* = 0.001; I^2^ = 29.3%) (S5 Fig).

#### Effect of complement-activating anti-HLA DSAs according to the timing of antibody detection

We performed a stratified analysis according to the time of antibody detection. We confirmed that in patients with either preexisting (HR 2.67; 95% CI 1.79–4.00, *P* = 0.001; I^2^ = 52.7%) anti-HLA DSAs or de novo (HR 3.65; 95% CI 2.45–5.44, *P* = 0.001; I^2^ = 38.0%) anti-HLA DSAs, complement-activating anti-HLA DSAs remained significantly associated with an increased risk of allograft loss (S6 Fig).

#### Analysis according to the type of test used for detecting complement-activating antibodies

Primary analyses were stratified according to the type of test used for detecting complement-activating antibodies. We found consistent associations across the different methods to detect complement-activating anti-HLA antibodies: (i) C1q-binding capacity (HR 2.80, 95% CI 2.11–3.71, I^2^ = 42.1%), (ii) IgG3 subclass (HR 3.11, 95% CI 2.29–4.22, I^2^ = 0.0%), (iii) C3d-binding capacity (HR 5.04, 95% CI 2.10–12.07, I^2^ = 51.2%), and (iv) C4d-binding capacity (HR 3.82, 95% CI 2.05–7.11, I^2^ = 29.8%). Because IgG3 subclass DSA may not provide the exact same information as complement-binding tests (C1q, C3d, C4d), we performed additional post hoc analyses and found similar associations when stratified according to complement-activating anti-HLA DSAs (C1q-, C3d-, and C4d-binding ability) and IgG subclass, with a pooled HR of 3.11 (95% CI 2.42–4.0, *P* = 0.001) and 3.11 (95% CI 2.29–4.22, *P* = 0.001), respectively (S7 Fig).

#### Center effect

After removing the 3 largest studies from the analysis [16,48,49], the presence of complement-activating anti-HLA DSAs remained significantly associated with an increased risk of allograft loss (HR 2.90; 95% CI 2.33–3.60, *P* = 0.001), and the heterogeneity across studies remained stable at 31.8% (S8 Fig).

In order to identify additional factors explaining residual heterogeneity, we performed metaregression and did not find any significant association between date of publication (*P* = 0.664), mean MFI for anti-HLA DSA (*P* = 0.632), number of HLA mismatch (*P* = 0.582), period of inclusion (*P* = 0.109), mean population age (*P* = 0.078), and the risk of allograft loss.

## Discussion

In the present meta-analysis including 7,936 solid organ transplant patients, we established that complement-activating anti-HLA DSAs represent an important determinant of allograft loss across multiple types of organ transplants without a significant publication bias and with acceptable heterogeneity. Patients with complement-activating anti-HLA DSAs have a 3-fold–increased risk of allograft loss compared with patients without anti-HLA DSAs and/or patients with non–complement-activating anti-HLA DSAs. These associations were consistent regarding long-term allograft loss in high-quality studies, across different solid organ transplant populations (kidney, heart, lung, and liver transplant recipients), across different types of tests used for detecting complement-activating anti-HLA DSAs, and at different times of evaluation for complement-activating anti-HLA DSA status (before and after transplantation). Moreover, beyond the effect on allograft survival, we found that complement-activating anti-HLA DSAs were also strongly associated with an increased risk of allograft rejection. These findings reinforce the robustness of the results and their applicability in different clinical scenarios and transplant programs with different practices and support the possibility of a causal effect between complement-activating antibodies and allograft injury.

One of the major hurdles in the quest to develop personalized medicine in transplantation and improve overall transplant patient outcomes is the lack of valid, mechanistically-informed noninvasive biomarkers for predicting allograft outcomes that can be used for patient risk stratification, clinical trial design, and as surrogate endpoints. The recognition of the dominant role of anti-HLA antibodies in rejection and late failure of kidney [10], heart [12], liver [13], lung [11], or intestinal [14] transplants has been a turning point for transplant medicine in the past decade. However, not all anti-HLA DSAs are equal in terms of pathogenicity and therefore may not be consistently associated with adverse allograft outcomes. Because activation of the complement cascade is an important component of the ABMR process, new approaches have been developed to better characterize anti-HLA DSAs and link their capacity to activate complement to the pathophysiology of transplant rejection. The complement-activating ability of anti-HLA antibodies and/or complement-activating IgG subclasses have been shown to be associated with more severe rejection episodes and diminished long-term graft survival [17,49,50]. However, some groups have reported different results, with varying magnitudes of effects ranging from strong to marginal associations between complement-activating anti-HLA DSAs and allograft loss [19,27].

The results of this meta-analysis were robust across diverse subgroup analyses. First, although kidney transplant patients represented the highest number of patients included in the present meta-analysis, the effects of complement-activating anti-HLA DSAs on allograft loss remained significant in heart, lung, and liver transplant patients. Grouping non-kidney transplant studies together (liver, lung, and heart transplantation) as opposed to kidney transplant studies was based on the larger volume of studies focusing on kidney transplant patients. This mirrors the distribution of solid organ transplants worldwide (84,347 kidney transplantations among the 126,670 total organs transplanted) [72].

Second, the same effect was observed regardless of whether the antibody was preexisting or de novo. Third, we found similar associations regardless of the type of test used for assessing complement-activating anti-HLA DSAs.

In most of the studies included in this meta-analysis, a correlation existed between complement-activating antibody status and anti-HLA DSA level (assessed by MFI). Despite this correlation, 8 studies included in the present meta-analysis with sufficient statistical power to perform multivariable models demonstrated that the association between C1q-, C3d-binding tests or IgG3 test and allograft outcomes was independent of the level of anti-HLA DSA MFI (Fig 5). Moreover, the SAB assays can be falsely low, while the C1q assay is more accurate. Therefore, the SAB assay has limitations that mislead the interpretation in comparing MFI versus C1q, C3d, or C4d assays [73]. In contrast to MFI that was reported in most of the studies in this meta-analysis, anti-HLA DSA level determined by titer of antibody correlated with complement-fixing ability [22,74]. In addition to the requirement of minimum titer of DSAs (>1:16) to be complement fixing, the composition of IgG subtypes may also influence the complement-binding capacity [48,75]. Therefore, C1q, C3d and IgG3 assays provide additional insights beyond the DSA strength/titer. Finally, the cutoffs used for antibody detection and for complement-activating anti-HLA DSAs in the different studies was variable. These different cutoffs and technical issues in anti-HLA DSA detection, such as avoidance of the prozone effect, are beyond the scope of the present study.

The heterogeneity (I^2^) found in the present study may be explained by (i) different tests and protocols used for screening complement-activating antibodies (C1q, C4d, C3d, and IgG subclass), (ii) different types of transplant cohorts and clinical management, including risk-taking strategies (high versus low immunological risk transplant populations), (iii) the timing of antibody detection before and after transplantation, and (iv) nonoptimal statistical power and statistical methodologies used in some studies. Despite this overall heterogeneity, when subgroup analyses were performed including studies with high methodological quality, the heterogeneity decreased from 29.3% to 3.1%. When patients with kidney transplantation were analyzed, the heterogeneity remained stable. Also, when studies using multivariable models were selected in the main analysis, the heterogeneity dropped to 17.4%. Last, despite the overall heterogeneity, the association between complement-activating antibodies and allograft loss remained highly significant in many different clinical scenarios, transplant populations, and relative to the timing of antibody detection, thereby reinforcing the study conclusions.

The findings of the present study have important clinical implications. The magnitude of the overall association found in the present study further reinforces the possibility of using circulating complement-activating anti-HLA DSAs as a potential prognostic factor for allograft loss in transplant patients. Relative to studies from other medical fields such as oncology or cardiology, well-recognized prognostic biomarkers did not always provide associations as high as the one observed in the present medical scenario [76–79]. Beyond their prognostic ability, the characterization of complement-activating anti-HLA DSA properties may influence the allocation system. The consolidation of the SAB–pan-IgG assay in the detection of preformed anti-HLA antibodies has improved transplantation success. However, its high sensitivity has limited the allograft allocation for sensitized patients. The result from this meta-analysis reveals that not all anti-HLA DSAs detected by SAB–pan-IgG assays are equally pathogenic, supporting that, overall, the neat-serum MFI value alone—which only offers a semiquantitative measurement of antibody level—is not entirely reliable for predicting transplant outcome. While the clinical use of SAB–C1q assay for the identification of unacceptable mismatches would improve wait-listed patient stratification regarding their risk of allograft loss, it might also increase the limited allograft allocation of highly sensitized patients—predefined by the standard SAB–pan-IgG assay but restratified as non–C1q-binding DSAs by the SAB–C1q assay—thereby shortening their waiting time.

Characterization of complement-activating anti-HLA DSAs may also have therapeutic significance, providing opportunities for the prevention and/or treatment of ABMR given the availability of specific drugs targeting complement or inhibiting complement-dependent cytotoxicity [80–82]. The present study provides an important step toward a pathogenesis-based approach for preventing and/or treating ABMR. Compared with the current approach to treatment, which only considers the presence of circulating anti-HLA DSAs, a risk-stratified approach on the basis of the complement-activating capacity of anti-HLA DSAs might significantly improve the response rate to complement-inhibitor drugs. The validity of this approach has recently been suggested in a clinical trial [83] in addition to post hoc analyses of 2 clinical trials (NCT01567085 and NCT01399593) including kidney transplant recipients with preformed anti-HLA DSAs receiving C5 inhibitor (eculizumab) for rejection prophylaxis, showing that the effect of eculizumab on allograft function depends on the complement-activating capacity of anti-HLA DSAs [84]. Further studies are needed for defining whether complement-activating anti-HLA DSAs have the potential to inform therapeutic decision-making for timely intervention and to streamline the use of expensive complement inhibitors in kidney transplantation.

We recognize the following limitations. We first acknowledge the higher proportion of kidney recipients compared to heart, liver, and lung transplant recipients. We also acknowledge that fewer studies regarding allograft rejection are included, which is partly due to the lack of histological phenotyping provided by the allograft biopsy in certain studies. Further studies are required to quantify the magnitude of the effect of complement-activating anti-HLA antibodies on the risk of allograft rejection and the efficacy of ABMR therapies. Third, the timing of anti-HLA detection is also a limitation, and because of the number of studies in the different groups of DSA detection, a comparison between groups was not reliable. Fourth, no data were available from Australian or South American transplant populations or from intestines or pancreas transplantation, limiting the extrapolation of our results to these patient populations. Finally, almost all of the included studies were observational and retrospective. Confounding factors from unknown origin may explain part of the residual heterogeneity observed.

In conclusion, circulating complement-activating anti-HLA DSAs represent a significant determinant of long-term allograft survival and solid organ transplant rejection and may be considered a potential valuable prognostic biomarker for improving the risk stratification for allograft loss.

## Supporting information

S1 PRISMA Checklist (DOC)

S1 Text Protocol of the research strategy. (DOCX)

S2 Text Search strategy in Ovid database. (DOCX)

S3 Text Quality assessment of nonrandomized trial. (DOCX)

S4 Text Supplementary references. (DOCX)

S1 Table Characteristics of the studies. (DOCX)

S2 Table Methodological quality assessment of nonrandomized trials. (DOCX)

S1 Fig Association of circulating complement-activating anti-HLA DSAs with the risk of allograft loss. Studies comparing complement-activating anti-HLA DSAs with non–complement-activating anti-HLA DSAs. Studies are listed by the date of publication. The black diamond-shaped boxes represent the HR for each individual study. The grey boxes around the black diamond represent the weight of the study, and lines represent the 95% CI for individual studies. The blue diamond at the end represents the overall HR. The number of patients in the overall population does not correspond to the sum of the different groups for the studies of Kaneku et al. (2012) (3 patients), Sicard et al. (2015) (4 patients), and Moktefi et al. (2017) (3 patients) either because the data for these patients were missing or because they were not involved in the analysis. CI, confidence interval; DSA, donor-specific antibody; HLA, human leukocyte antigen; HR, hazard ratio.
(TIFF)

S2 Fig Association of circulating complement-activating anti-HLA DSAs with the risk of allograft loss. Studies comparing complement-activating anti-HLA DSAs with a mixed group of patients without anti-HLA DSAs and with non–complement-activating anti-HLA DSAs. Studies are listed by the date of publication. The black diamond-shaped boxes represent the HR for each individual study. The grey boxes around the black diamond represent the weight of the study, and lines represent the 95% CI for individual studies. The blue diamond at the end represents the overall HR. CI, confidence interval; DSA, donor-specific antibody; HLA, human leukocyte antigen; HR, hazard ratio.
(TIFF)

S3 Fig Association between complement-activating anti-HLA DSAs and the risk of rejection. Studies comparing complement-activating anti-HLA DSAs with non–complement-activating anti-HLA DSAs. Studies are listed by date of publication. The black diamond-shaped boxes represent the HR for each individual study. The grey boxes around the black diamond represent the weight of the study, and lines represent the 95% CI for individual studies. The blue diamond at the end represents the overall HR. CI, confidence interval; DSA, donor-specific antibody; HLA, human leukocyte antigen; HR, hazard ratio.
(TIFF)

S4 Fig Association of circulating complement-activating anti-HLA DSAs with the risk of allograft loss including only high–methodological quality studies. Studies are listed by date of publication. The black diamond-shaped boxes represent the HR for each individual study. The grey boxes around the black diamond represent the weight of the study, and lines represent the 95% CI for individual studies. The blue diamond at the end represents the overall HR. The number of patients in the overall population does not correspond to the sum of the different groups for the studies of Kaneku et al. (2012) (3 patients) and Sicard et al. (2015) (4 patients) either because the data for these patients were missing or because they were not involved in the analysis. CI, confidence interval; DSA, donor-specific antibody; HLA, human leukocyte antigen; HR, hazard ratio.
(TIFF)

S5 Fig Association of circulating complement-activating anti-HLA DSAs with the risk of allograft loss stratified by the type of transplanted organ. Studies are listed by date of publication. The black diamond-shaped boxes represent the HR for each individual study. The grey boxes around the black diamond represent the weight of the study, and lines represent the 95% CI for individual studies. The blue diamond at the end represents the overall HR. Number of patients in the overall population does not correspond to the sum of the different groups for the studies of Kaneku et al. (2012) (3 patients), Sicard et al. (2015) (4 patients), and Moktefi et al. (2017) (3 patients) either because the data for these patients were missing or because they were not involved in the analysis. CI, confidence interval; DSA, donor-specific antibody; HLA, human leukocyte antigen; HR, hazard ratio.
(TIFF)

S6 Fig Association of circulating complement-activating anti-HLA DSAs with the risk of allograft loss stratified by the timing of anti-HLA DSA detection. Studies are listed by date of publication. The black diamond-shaped boxes represent the HR for each individual study. The grey boxes around the black diamond represent the weight of the study, and lines represent the 95% CI for individual studies. The blue diamond at the end represents the overall HR. Number of patients in the overall population does not correspond to the sum of the different groups for the studies of Kaneku et al. (2012) (3 patients), Sicard et al. (2015) (4 patients), and Moktefi et al. (2017) (3 patients) either because the data for these patients were missing or because they were not involved in the analysis. CI, confidence interval; DSA, donor-specific antibodies; HLA, human leukocyte antigen; HR, hazard ratio.
(TIFF)

S7 Fig Association of circulating complement-activating anti-HLA DSAs with the risk of allograft loss according to the type of test used to determine complement-activating antibody capacity, either complement-binding antibody or IgG3 subclass. Studies are listed by date of publication. The black diamond-shaped boxes represent the HR for each individual study. The grey boxes around the black diamond represent the weight of the study, and lines represent the 95% CI for individual studies. The blue diamond at the end represents the overall HR. The number of patients in the overall population does not correspond to the sum of the different groups for the studies of Kaneku et al. (2012) (3 patients), Sicard et al. (2015) (4 patients), and Moktefi et al. (2017) (3 patients) either because the data for these patients were missing or because they were not involved in the analysis. CI, confidence interval; DSA, donor-specific antibody; HLA, human leukocyte antigen; HR, hazard ratio.
(TIFF)

S8 Fig Association of circulating complement-activating anti-HLA DSAs with the risk of allograft loss after exclusion of the 3 largest studies [16,48,49]. Studies are listed by the date of publication. The black diamond-shaped boxes represent the HR for each individual study. The grey boxes around the black diamond represent the weight of the study, and lines represent the 95% CI for individual studies. The blue diamond at the end represents the overall HR. The number of patients in the overall population does not correspond to the sum of the different groups for the studies of Kaneku et al. (2012) (3 patients), Sicard et al. (2015) (4 patients), and Moktefi et al. (2017) (3 patients) either because the data for these patients were missing or because they were not involved in the analysis. CI, confidence interval; DSA, donor-specific antibody; HLA, human leukocyte antigen; HR, hazard ratio.
(TIFF)

## Abbreviations

ABMR: antibody-mediated rejection
C1q: complement component 1q
CI: confidence interval
DSA: donor-specific antibody
HLA: human leukocyte antigen
HR: hazard ratio
IgG3: immunoglobulin G3
ISHLT: International Society for Heart and Lung Transplantation
IVIg: intravenous immunoglobulin
MFI: mean fluorescence intensity
MOOSE: Meta-Analyses of Observational Studies in Epidemiology
NC: not communicated
NOS: Newcastle–Ottawa Scale
OR: odds ratio
PP: plasmapheresis
PRISMA: Preferred Reporting Items for Systematic Reviews and Meta-Analyses
SAB: single-antigen bead
SE: standard error
