# Correction: Complement-activating donor-specific anti-HLA antibodies and solid organ transplant survival: A systematic review and meta-analysis

**DOI:** 10.1371/journal.pmed.1002637

**Published:** 2018-07-27

**Authors:** Antoine Bouquegneau, Charlotte Loheac, Olivier Aubert, Yassine Bouatou, Denis Viglietti, Jean–Philippe Empana, Camilo Ulloa, Mohammad Hassan Murad, Christophe Legendre, Denis Glotz, Annette M. Jackson, Adriana Zeevi, Stephan Schaub, Jean–Luc Taupin, Elaine F. Reed, John J. Friedewald, Dolly B. Tyan, Caner Süsal, Ron Shapiro, E. Steve Woodle, Luis G. Hidalgo, Jacqueline O’Leary, Robert A. Montgomery, Jon Kobashigawa, Xavier Jouven, Patricia Jabre, Carmen Lefaucheur, Alexandre Loupy

The eighth author’s name was entered into the submission system incorrectly and therefore was indexed incorrectly on PubMed. The correct name is Mohammad Hassan Murad. The correct indexing in PubMed should read Murad MH.
