# Supplementary material for: Complement-activating donor-specific anti-HLA antibodies and solid organ transplant survival: A systematic review and meta-analysis
Source: PLoS Med. 2018 May 25;15(5):e1002572. doi: 10.1371/journal.pmed.1002572 (PMC5969739; doi:10.1371/journal.pmed.1002572)
Supplement: S1 Text — (DOCX) [file pmed.1002572.s002.docx]

***Protocol of the research strategy***

*Review question*

Is there a relation between complement-activating anti-human leucocyte antigen donor specific antibodies and graft survival or episodes of rejection in solid organ transplant patients?

*Specific aims*

Aim 1)

To assess the impact of complement-activating anti-Human Leukocyte Antigen (HLA) donor specific antibodies (DSA) on the death censored allograft survival in solid organ transplant patients, by systematically reviewing studies looking for this specific outcome.

Aim 2)

To evaluate the impact of complement-activating anti-HLA DSA on the risk of biopsy proven rejection episode.

**Hypothesis:**

Circulating complement-activating anti-HLA DSAs are associated with a lower graft survival and an increased risk of rejection compared to patients with circulating anti-HLA DSAs without the capability of activating the complement.

**Relevance of the proposed research:**

This meta-analysis aims to tackle the controversy on the role of complement activating anti-HLA DSAs and the graft survival or rejection appearance, by reviewing in an exhaustive way, all the studies that evaluated the impact of each complement activating anti-HLA DSA or IgG subclass on this specific outcome. Depending on findings, the integration of the assessment of complement activating anti-HLA DSAs in the patient management and also guide the treatment.

*Eligibility criteria*

**Inclusion:**

Study designs:
Case-crossover study, case control study, cohort study, randomized control study and of any language.

Participants:

Solid organ transplant population: kidney, heart, liver, lung and intestines (small bowel) transplant recipients.

Either adult or paediatric patients.

Measurement:

Donor specific anti-human leukocyte antigen antibodies by luminex solid-phase assay.

Complement activating anti-HLA DSA (C1q, C3d, C4d) and/or IgG subclass.

Outcomes of interest:

Graft survival/loss and/or acute rejection.

**EXCLUSION**

Animal, *ex vivo* and methodological studies.

*Search Strategy*

**Years:** data base inception to January 31th, 2018

**Databases:** Ovid MEDLINE In-Process & Other Non-Indexed Citations, Ovid MEDLINE, Ovid EMBASE, Ovid Cochrane Central Register of Controlled Trials, Ovid Cochrane Database of Systematic Reviews, and Scopus

**Search terms:** “solid organ transplantation” “kidney transplantation” “liver transplantation” “lung transplantation” “heart transplantation” “small bowel / intestine transplantation” “donor specific antibodies” “HLA DSA” “single-antigen bead array” “complement binding DSA” “complement activating DSA” “C1q” “C3d” “C4d” “IgG subclass” “outcome” “graft survival “graft loss” “antibody-mediated rejection” “acute rejection” “chronic rejection”.

In addition, we will review the reference sections of eligible studies and available reviews. We also request potentially eligible studies from content experts.

*Selection*

Two reviewers (C.Loheac and A. Bouquegneau) will consider the potential eligibility of each of the abstracts and titles that result from executing the search strategy. Reviewers will request the full text versions of all potentially eligible studies. Disagreements will also be retrieved in full text for evaluation with C.Lefaucheur and A. Loupy.

Two reviewers (C.Loheac and A. Bouquegneau) working independently and blindly will consider the full text reports (all available versions of each study) for eligibility. The reviewers will calibrate their judgments using a smaller set of reports. Subsequently, disagreements will be harmonized by consensus; if not possible, by arbitration by C.Lefaucheur and A. Loupy. Agreement will be measured using the kappa or phi statistics, as appropriate (the latter is appropriate when the distribution of agreement is extreme).

*Extraction*

Data extraction will include details from study population, author name, year of publication, study size, mean or median follow-up time, type of complement-activating anti-HLA DSAs, comparison used (patients with complement-activating anti-HLA DSAs compared either to patients without complement-activating DSAs, patients without DSAs detected, or a mixed group of patients without DSAs and without complement-activating DSAs), effect sizes (HR and/or OR), 95% confidence intervals (CIs), potential confounding factors, and unadjusted and adjusted estimated risks of graft loss or graft rejection. Adjusted HRs and ORs will be used when available; otherwise, univariate effect sizes will be used.

*Quality*

To assess the methodological quality of studies, we will determine:

- The use of single-antigen flow bead assays to detect donor-specific anti-HLA antibodies and complement-activating DSA
- The level of mean fluorescence intensity (MFI) considered as positive
- Time of follow up
- Validated criteria of rejection according to Banff [1,2] or ISHLT classification [3,4]
- Allograft loss have been or not adjusted/censored for death
- The presence of multi-varied analysis and potential confounders factors

Finally, we will ensure that items of the Newcastle-Ottawa quality assessment Scale (NOS) will be verified to quantify studies quality for observational studies [5]. Jadad scale will be used to assess a quality of randomized controlled trials [6].

*Pooling*

When possible, we will generate meta-analytic estimates of each complement activating DSA types on the occurrence of graft loss and rejection (relative risk, odd-ratio and percentage change). As a rule we will use random-effects meta-analyses and measure inconsistency for each outcome by estimating the I^2^ test and its confidence interval [7]. We will use several available software packages to conduct the analyses (STATA software version 14.1, R software version 3.2.1).

*Subgroups*

To explore causes of inconsistency and subgroup-confounders interactions, we will construct the following subgroup analyses defined by:

1. **Comparator group used:** studies in which a comparison chosen will be between index group (complement-activating anti-HLA DSA) and patients without complement activating anti-HLA will be analysed separately than studies comparing index group with a mixed group of patients without complement-activating anti-HLA DSAs and without anti-HLA DSAs
2. **Studies that used multivariable models** for addressing the independent associations of complement activation with allograft failure
3. **High methodological quality studies** (NOS scores ≥6 ) and **low methodological quality** (NOS scores <6) [8]
4. **Type of organ transplanted:** kidney allograft and all other types of transplanted organs.
5. **Timing of antibody detection:** pre-existing anti-HLA DSAs, defined as antibodies present before or at the time of transplantation, or *de novo* anti-HLA DSAs, defined as antibodies present only after transplantation, or a combined group of pre-existing and *de novo* DSAs
6. **Type of assay used for characterizing the complement-activating capacity of antibodies:** assays were characterised as anti-HLA DSA IgG subclass, C1q-binding anti-HLA DSAs, C4d-binding anti-HLA DSAs, or C3d-binding anti-HLA DSAs.
7. **Centre effect:** this subgroup analysis excluded the largest cohorts (in terms of the number of patients included) [9–11]. Larger studies could dominate the overall results of the meta-analysis and could therefore decrease heterogeneity.

We will measure the difference in effect sizes between subgroups (univariate analyses). When possible, we will construct meta-regression analyses with subgroups as the independent variables and outcomes as the dependent variable. Meta-regression, is an extension to standard meta-analysis that investigates to which extent statistical heterogeneity between results of multiple studies can be associated with one or more characteristics of the studies [12]. It is described as the merging of meta-analytic techniques with linear regression principles (predicting treatment effects using covariates). We adjusted effects sizes on use of trial-level covariates, such as date of publication, mean fluorescence intensity (MFI) for anti-HLA DSA detection, and mean population age. We used the overall model p-value to assess if there is evidence for an association of any of the covariates with the outcome [13].

Meta-regression could also explore possible causes of heterogeneity and ascertain stability of results between subgroup analysis.

*Sensitivity Analyses*

When relevant we will explore how results of the meta-analyses change when using fixed effects models [14], when borderline eligible studies are included or excluded. The center effect will also be studied thank to the exclusion of the larger studies.

*Reporting heterogeneity and bias*

Statistical heterogeneity across the studies will be tested with the I^2^ index [15]. The I^2^ index describes the percentage of total variation across studies due to heterogeneity rather than chance. A value of 0% indicates no observed heterogeneity, values exceeding 50% may elicit considerable caution and warrant further analysis through subgroup analyses [16]. A low p-value of the I^2^ test (below 0.05) provides evidence of heterogeneity of intervention effects (variation in effect estimates beyond chance).

Publication bias will be visually assessed using funnel plots and statistically assessed by the Egger’s bias coefficient, which weighted the regression of the intervention effect on its standard error (SE), with weights inversely proportional to the variance of the intervention effect [17]. A p-value < 0.05 (2-sided) was considered statistically significant for the presence of a publication bias.

We will contact authors of the included studies if needed for clarification.
