# Supplementary material for: Complement-activating donor-specific anti-HLA antibodies and solid organ transplant survival: A systematic review and meta-analysis
Source: PLoS Med. 2018 May 25;15(5):e1002572. doi: 10.1371/journal.pmed.1002572 (PMC5969739; doi:10.1371/journal.pmed.1002572)
Supplement: S2 Text — (DOCX) [file pmed.1002572.s003.docx]

*Search strategy in Ovid database*

Database: Ovid MEDLINE(R) Epub Ahead of Print, In-Process & Other Non-Indexed Citations, Ovid MEDLINE(R) Daily and Ovid MEDLINE(R) 1946 to Present

|  | **Searches** | **Results** |
| --- | --- | --- |
| **1** | exp kidney transplantation/ | **207779** |
| **2** | ((kidney* or renal) adj3 (transplant* or graft* or allotransplant* or "allo-transplant*" or homotransplant* or "homo-transplant*" or retransplant* or "re-transplant*" or autotransplant* or "auto-transplant*" or allograft* or "allo-graft*" or homograft* or "homo-graft*" or "cadaver kidney*")).mp. | **249054** |
| **3** | exp Liver Transplantation/ | **147392** |
| **4** | ((liver or hepatic) adj3 (transplant* or graft* or allotransplant* or "allo-transplant*" or homotransplant* or "homo-transplant*" or retransplant* or "re-transplant*" or autotransplant* or "auto-transplant*" or allograft* or "allo-graft*" or homograft* or "homo-graft*" or "cadaver liver*")).mp. | **180011** |
| **5** | exp Heart Transplantation/ | **86225** |
| **6** | ((heart or cardiac) adj3 (transplant* or graft* or allotransplant* or "allo-transplant*" or homotransplant* or "homo-transplant*" or retransplant* or "re-transplant*" or autotransplant* or "auto-transplant*" or allograft* or "allo-graft*" or homograft* or "homo-graft*" or "cadaver heart*" or heterotransplant* or "hetero-transplant*")).mp. | **112895** |
| **7** | exp Lung Transplantation/ | **44160** |
| **8** | ((lung or pulmonary) adj3 (transplant* or graft* or allotransplant* or "allo-transplant*" or homotransplant* or "homo-transplant*" or retransplant* or "re-transplant*" or autotransplant* or "auto-transplant*" or allograft* or "allo-graft*" or homograft* or "homo-graft*" or "cadaver lung*" or heterotransplant* or "hetero-transplant*")).mp. | **60521** |
| **9** | exp Intestines/tr [Transplantation] | **8428** |
| **10** | ileum graft/ or exp intestine graft/ | **2007** |
| **11** | ((intestin* or "peritoneum free" or ileum or ileac or "small bowel*") adj3 (transplant* or graft* or allotransplant* or "allo-transplant*" or homotransplant* or "homo-transplant*" or retransplant* or "re-transplant*" or autotransplant* or "auto-transplant*" or allograft* or "allo-graft*" or homograft* or "homo-graft*" or heterotransplant* or "hetero-transplant*")).mp. | **12529** |
| **12** | (("solid organ" or "solid organs") adj3 (transplant* or graft* or allotransplant* or "allo-transplant*" or homotransplant* or "homo-transplant*" or retransplant* or "re-transplant*" or autotransplant* or "auto-transplant*" or allograft* or "allo-graft*" or homograft* or "homo-graft*" or "cadaver organ*" or heterotransplant* or "hetero-transplant*")).mp. | **21162** |
| **13** | or/1-12 | **575199** |
| **14** | ((("complement activating" or "complement binding") adj5 (DSA or DSAs or "donor specific" or antibod* or "anti-bod*" or alloantibod* or "alloanti-bod*" or "allo-antibod*" or "allo-anti-bod*")) or (("donor specific" or "donor HLA-specific" or "human leukocyte antigen*" or "anti-HLA" or antiHLA or HLA) adj5 (DSA or DSAs or antibod* or "anti-bod*" or alloantibod* or "alloanti-bod*" or "allo-antibod*" or "allo-anti-bod*")) or "antiHLA DSA" or "antiHLA DSAs" or C1q or C3d or C4d or "HLA DSA" or "HLA DSAs" or "IgG subclass*" or "IgG1 subclass*" or "IgG3 subclass*" or "IgG4 subclass*" or "immunoglobulin G1 subclass*" or "immunoglobulin G3 subclass*" or "immunoglobulin G4 subclass*" or "immunoglobulin-G subclass*" or "single-antigen bead array*").mp. | **59129** |
| **15** | 13 and 14 | **14306** |
| **16** | exp Treatment Outcome/ | **2291524** |
| **17** | exp Graft Rejection/ | **152802** |
| **18** | exp Graft Survival/ | **101865** |
| **19** | ("allograft reaction*" or "allo-graft reaction*" or fail* or "graft reaction*" or "homograft reaction*" or "homo-graft reaction*" or "homotransplant reaction*" or "homo-transplant reaction*" or "host defense reaction*" or "host donor cell interaction*" or "host graft response*" or "host versus graft reaction*" or "host versus graft syndrome*" or loss or outcome* or reject* or surviv*).mp. | **10264341** |
| **20** | 16 or 17 or 18 or 19 | **10266842** |
| **21** | 15 and 20 | **12856** |
| **22** | exp evidence based medicine/ | **1077022** |
| **23** | exp meta analysis/ | **220146** |
| **24** | exp Meta-Analysis as Topic/ | **53749** |
| **25** | exp "systematic review"/ | **148529** |
| **26** | exp Guideline/ or exp Practice Guideline/ | **456306** |
| **27** | exp controlled study/ | **5905315** |
| **28** | exp Randomized Controlled Trial/ | **935938** |
| **29** | exp triple blind procedure/ | **172** |
| **30** | exp Double-Blind Method/ | **407396** |
| **31** | exp Single-Blind Method/ | **71839** |
| **32** | exp latin square design/ | **342** |
| **33** | exp Placebos/ | **326963** |
| **34** | exp Placebo Effect/ | **10077** |
| **35** | exp comparative study/ | **2831735** |
| **36** | exp Cross-Sectional Studies/ | **494986** |
| **37** | exp Cross-Over Studies/ | **128296** |
| **38** | exp Cohort Studies/ | **2206266** |
| **39** | exp longitudinal study/ | **341179** |
| **40** | exp retrospective study/ | **1253366** |
| **41** | exp prospective study/ | **953855** |
| **42** | exp observational study/ | **168907** |
| **43** | exp clinical trial/ | **2033082** |
| **44** | clinical study/ | **109307** |
| **45** | in vivo study/ | **277375** |
| **46** | exp correlational study/ | **26516** |
| **47** | exp case-control studies/ | **1064678** |
| **48** | exp confidence interval/ | **165725** |
| **49** | exp regression analysis/ | **775838** |
| **50** | exp proportional hazards model/ | **146845** |
| **51** | exp multivariate analysis/ | **462683** |
| **52** | ((meta adj analys*) or (systematic* adj3 review*) or guideline* or (control* adj3 study) or (control* adj3 trial) or (randomized adj3 study) or (randomized adj3 trial) or (randomised adj3 study) or (randomised adj3 trial) or "pragmatic clinical trial" or (doubl* adj blind*) or (doubl* adj mask*) or (singl* adj blind*) or (singl* adj mask*) or (tripl* adj blind*) or (tripl* adj mask*) or (trebl* adj blind*) or (trebl* adj mask*) or "latin square" or placebo* or nocebo* or "comparative study" or "comparative survey" or "comparative analysis" or "cross-sectional study" or "cross-sectional analysis" or "cross-sectional survey" or "cross-sectional design" or "prevalence study" or "prevalence analysis" or "prevalence survey" or "disease frequency study" or "disease frequency analysis" or "disease frequency survey" or crossover or "cross-over" or cohort* or "longitudinal study" or "longitudinal survey" or "longitudinal analysis" or "longitudinal evaluation" or longitudinal* or ((retrospective or "ex post facto") adj3 (study or survey or analysis or design)) or retrospectiv* or "prospective study" or "prospective survey" or "prospective analysis" or prospectiv* or (("follow-up" or followup) adj (stud* or survey or analysis)) or ((observation or observational) adj (study or survey or analysis)) or "clinical study" or "clinical trial" or "in vivo study" or "in vivo analysis" or ((correlation* adj2 study) or (correlation* adj2 analys*)) or "case control study" or "case base study" or "case referrent study" or "case referent study" or "case referent study" or "case compeer study" or "case comparison study" or "matched case control" or "multicenter study" or "multi-center study" or "odds ratio" or "confidence interval" or "regression analysis" or "least square" or "least squares" or (hazard* adj (model* or analys* or regression or ratio or ratios)) or "Cox model" or "Cox multivariate analyses" or "Cox multivariate analysis" or "Cox regression" or "Cox survival analyses" or "Cox survival analysis" or "Cox survival model" or "change analysis").mp,pt. | **16682167** |
| **53** | or/22-52 | **17201711** |
| **54** | 21 and 53 | **7217** |
| **55** | from 21 keep 9031-12856 | **3826** |
| **56** | limit 55 to (clinical study or clinical trial, all or clinical trial, phase i or clinical trial, phase ii or clinical trial, phase iii or clinical trial, phase iv or clinical trial or controlled clinical trial or multicenter study or observational study or randomized controlled trial or pragmatic clinical trial or comparative study or controlled clinical trial or guideline or practice guideline or meta analysis or multicenter study or observational study or randomized controlled trial or pragmatic clinical trial or systematic reviews) [Limit not valid in Embase,CCTR,CDSR; records were retained] | **514** |
| **57** | 54 or 56 | **7232** |
| **58** | (exp animals/ or exp nonhuman/) not exp humans/ | **9118728** |
| **59** | ((alpaca or alpacas or amphibian or amphibians or animal or animals or antelope or armadillo or armadillos or avian or baboon or baboons or beagle or beagles or bee or bees or bird or birds or bison or bovine or buffalo or buffaloes or buffalos or "c elegans" or "Caenorhabditis elegans" or camel or camels or canine or canines or carp or cats or cattle or chick or chicken or chickens or chicks or chimp or chimpanze or chimpanzees or chimps or cow or cows or "D melanogaster" or "dairy calf" or "dairy calves" or deer or dog or dogs or donkey or donkeys or drosophila or "Drosophila melanogaster" or duck or duckling or ducklings or ducks or equid or equids or equine or equines or feline or felines or ferret or ferrets or finch or finches or fish or flatworm or flatworms or fox or foxes or frog or frogs or "fruit flies" or "fruit fly" or "G mellonella" or "Galleria mellonella" or geese or gerbil or gerbils or goat or goats or goose or gorilla or gorillas or hamster or hamsters or hare or hares or heifer or heifers or horse or horses or insect or insects or jellyfish or kangaroo or kangaroos or kitten or kittens or lagomorph or lagomorphs or lamb or lambs or llama or llamas or macaque or macaques or macaw or macaws or marmoset or marmosets or mice or minipig or minipigs or mink or minks or monkey or monkeys or mouse or mule or mules or nematode or nematodes or octopus or octopuses or orangutan or "orang-utan" or orangutans or "orang-utans" or oxen or parrot or parrots or pig or pigeon or pigeons or piglet or piglets or pigs or porcine or primate or primates or quail or rabbit or rabbits or rat or rats or reptile or reptiles or rodent or rodents or ruminant or ruminants or salmon or sheep or shrimp or slug or slugs or swine or tamarin or tamarins or toad or toads or trout or urchin or urchins or vole or voles or waxworm or waxworms or worm or worms or xenopus or "zebra fish" or zebrafish) not (human or humans or patient or patients)).ti,ab,hw,kw. | **7843211** |
| **60** | 57 not (58 or 59) | **7009** |
| **61** | limit 60 to (editorial or erratum or letter or note or addresses or autobiography or bibliography or biography or blogs or comment or dictionary or directory or interactive tutorial or interview or lectures or legal cases or legislation or news or newspaper article or overall or patient education handout or periodical index or portraits or published erratum or video-audio media or webcasts) [Limit not valid in Embase,CCTR,CDSR,Ovid MEDLINE(R),Ovid MEDLINE(R) Daily Update,Ovid MEDLINE(R) In-Process,Ovid MEDLINE(R) Publisher; records were retained] | **78** |
| **62** | from 61 keep 55-62 | **8** |
| **63** | (60 not 61) or 62 | **6939** |
| **64** | limit 63 to yr="2011 -Current" | **4618** |
| **65** | remove duplicates from 64 | **3555** |
| **66** | 63 not 64 | **2321** |
| **67** | remove duplicates from 66 | **1655** |
| **68** | 65 or 67 | **5210** |
