# Supplementary material for: Complement-activating donor-specific anti-HLA antibodies and solid organ transplant survival: A systematic review and meta-analysis
Source: PLoS Med. 2018 May 25;15(5):e1002572. doi: 10.1371/journal.pmed.1002572 (PMC5969739; doi:10.1371/journal.pmed.1002572)
Supplement: S3 Text — (DOCX) [file pmed.1002572.s004.docx]

***Quality assessment of non-randomized trial***

The Newcastle-Ottawa Scale (NOS) was used to assess the quality of the non-randomized studies included in the systematic review and meta-analysis [5]. Using this quality score, each study is judged on eight items categorized into the following 3 major components: selection of the study groups and ascertainment (0 to 4 points), quality of the adjustments for confounding variables (0 to 2 points), and outcomes (0 to 3 points). Stars awarded for each quality item serve as a quick visual assessment. Stars are awarded such that the highest quality studies are awarded up to nine stars. A higher score represents better methodological quality.

The 3 components are described below:

1. *Selection of the study groups*
   1. **Representativeness of the exposed cohort**
      1. Truly representative of the average in the community *****
      2. Somewhat representative of the average in the community *****
      3. Selected group of users, e.g., nurses, volunteers
      4. No description of the derivation of the cohort
   2. **Selection of the non-exposed cohort**
      1. Drawn from the same community as the exposed cohort *****
      2. Drawn from a different source
      3. No description of the derivation of the non-exposed cohort
   3. **Ascertainment of exposure**
      1. Secure record (e.g., surgical records) *****
      2. Structured interview *****
      3. Written self-report
      4. No description
   4. **Demonstration that the outcome of interest was not present at the start of the study** (for example, surveillance biopsy in cases of rejection evaluation)
      1. Yes*****
      2. No
2. *Comparability*
   1. **Comparability of the cohorts on the basis of the design or analysis**
      1. Study controls for certain statistical parameters (over-fitting of statistical models, not enough events, variables included in multivariate models) *****
      2. Study controls for any additional factors ***** (description for not using multivariate models)
3. *Outcomes*
   1. **Assessment of outcomes**
      1. Independent blind assessment *****
      2. Record linkage *****
      3. Self-report
      4. No description
   2. **Sufficient follow-up for outcomes**
      1. Yes (5-years) *****
      2. No
   3. **Adequacy of the follow-ups for cohorts**
      1. Complete follow-up - all subjects accounted for *****
      2. Subjects lost to follow-up who are unlikely to introduce bias – a small number lost *****
      3. Follow up rate < 80% and no description of those lost
      4. No statement
