# Supplementary material for: Complement-activating donor-specific anti-HLA antibodies and solid organ transplant survival: A systematic review and meta-analysis
Source: PLoS Med. 2018 May 25;15(5):e1002572. doi: 10.1371/journal.pmed.1002572 (PMC5969739; doi:10.1371/journal.pmed.1002572)
Supplement: S4 Text — (DOCX) [file pmed.1002572.s005.docx]

**Supplementary references**

1. Loupy A, Haas M, Solez K, Racusen L, Glotz D, Seron D, et al. The Banff 2015 Kidney Meeting Report: Current Challenges in Rejection Classification and Prospects for Adopting Molecular Pathology. Am J Transplant. 2017;17: 28–41. doi:10.1111/ajt.14107

2. Demetris A, Adams D, Bellamy C, Blakolmer K, Clouston A, Dhillon AP, et al. Update of the International Banff Schema for Liver Allograft Rejection: working recommendations for the histopathologic staging and reporting of chronic rejection. An International Panel. Hepatology. 2000;31: 792–799. doi:10.1002/hep.510310337

3. Berry GJ, Angelini A, Burke MM, Bruneval P, Fishbein MC, Hammond E, et al. The ISHLT working formulation for pathologic diagnosis of antibody-mediated rejection in heart transplantation: evolution and current status (2005-2011). J Heart Lung Transplant. 2011;30: 601–611. doi:10.1016/j.healun.2011.02.015

4. Meyer KC, Raghu G, Verleden GM, Corris PA, Aurora P, Wilson KC, et al. An international ISHLT/ATS/ERS clinical practice guideline: Diagnosis and management of bronchiolitis obliterans syndrome. Eur Respir J. 2014;44: 1479–1503. doi:10.1183/09031936.00107514

5. Wells GA, Shea B, O’Connell D, Peterson J, Welch V, Losos M, et al. The Newcastle-Ottawa Scale (NOS) for assessing the quality of non- randomised studies in meta-analyses. Available from: http://www.ohri.ca/programs/ clinical_epidemiology/oxford.htm.

6. Jadad AR, Moore RA, Carroll D, Jenkinson C, Reynolds DJ, Gavaghan DJ, et al. Assessing the quality of reports of randomized clinical trials: is blinding necessary? Control Clin Trials. 1996;17: 1–12.

7. Nikolakopoulou A, Mavridis D, Salanti G. Demystifying fixed and random effects meta-analysis. Evid Based Ment Health. 2014;17: 53–57. doi:10.1136/eb-2014-101795

8. McPheeters ML, Kripalani S, Peterson NB, Idowu RT, Jerome RN, Potter SA, et al. Closing the quality gap: revisiting the state of the science (vol. 3: quality improvement interventions to address health disparities). Evid ReportTechnology Assess. 2012;208.3: 1–475.

9. Loupy A, Lefaucheur C, Vernerey D, Prugger C, Duong van Huyen J-P, Mooney N, et al. Complement-binding anti-HLA antibodies and kidney-allograft survival. N Engl J Med. 2013;369: 1215–1226. doi:10.1056/NEJMoa1302506

10. Lefaucheur C, Viglietti D, Bentlejewski C, Huyen J-PD van, Vernerey D, Aubert O, et al. IgG Donor-Specific Anti-Human HLA Antibody Subclasses and Kidney Allograft Antibody-Mediated Injury. J Am Soc Nephrol. 2016;27: 293–304. doi:10.1681/ASN.2014111120

11. Viglietti D, Loupy A, Vernerey D, Bentlejewski C, Gosset C, Aubert O, et al. Value of Donor–Specific Anti–HLA Antibody Monitoring and Characterization for Risk Stratification of Kidney Allograft Loss. J Am Soc Nephrol. 2017;28: 702–715. doi:10.1681/ASN.2016030368

12. Thompson SG, Higgins JPT. How should meta-regression analyses be undertaken and interpreted? Stat Med. 2002;21: 1559–1573. doi:10.1002/sim.1187

13. Harbord RM, Higgins JPT. Meta-regression in Stata. Stata J. 2008;4: 493–519.

14. Nikolakopoulou A, Mavridis D, Salanti G. How to interpret meta-analysis models: fixed effect and random effects meta-analyses. Evid Based Ment Health. 2014;17: 64. doi:10.1136/eb-2014-101794

15. Higgins JPT, Thompson SG, Deeks JJ, Altman DG. Measuring inconsistency in meta-analyses. BMJ. 2003;327: 557–560. doi:10.1136/bmj.327.7414.557

16. Higgins JPT, Thompson SG. Quantifying heterogeneity in a meta-analysis. Stat Med. 2002;21: 1539–1558. doi:10.1002/sim.1186

17. Egger M, Davey Smith G, Schneider M, Minder C. Bias in meta-analysis detected by a simple, graphical test. BMJ. 1997;315: 629–634.

18. Wahrmann M, Bartel G, Exner M, Regele H, Körmöczi GF, Fischer GF, et al. Clinical relevance of preformed C4d-fixing and non-C4d-fixing HLA single antigen reactivity in renal allograft recipients. Transpl Int. 2009;22: 982–989. doi:10.1111/j.1432-2277.2009.00912.x

19. Hönger G, Wahrmann M, Amico P, Hopfer H, Böhmig GA, Schaub S. C4d-fixing capability of low-level donor-specific HLA antibodies is not predictive for early antibody-mediated rejection. Transplantation. 2010;89: 1471–1475. doi:10.1097/TP.0b013e3181dc13e7

20. Sutherland SM, Chen G, Sequeira FA, Lou CD, Alexander SR, Tyan DB. Complement-fixing donor-specific antibodies identified by a novel C1q assay are associated with allograft loss. Pediatr Transplant. 2012;16: 12–17. doi:10.1111/j.1399-3046.2011.01599.x

21. Hönger G, Hopfer H, Arnold M-L, Spriewald BM, Schaub S, Amico P. Pretransplant IgG subclasses of donor-specific human leukocyte antigen antibodies and development of antibody-mediated rejection. Transplantation. 2011;92: 41–47. doi:10.1097/TP.0b013e31821cdf0d

22. Smith JD, Banner NR, Hamour IM, Ozawa M, Goh A, Robinson D, et al. De novo donor HLA-specific antibodies after heart transplantation are an independent predictor of poor patient survival. Am J Transplant. 2011;11: 312–319. doi:10.1111/j.1600-6143.2010.03383.x

23. Kaneku H, O’Leary JG, Taniguchi M, Susskind BM, Terasaki PI, Klintmalm GB. Donor-specific human leukocyte antigen antibodies of the immunoglobulin G3 subclass are associated with chronic rejection and graft loss after liver transplantation. Liver Transpl. 2012;18: 984–992. doi:10.1002/lt.23451

24. Bartel G, Wahrmann M, Schwaiger E, Kikic Z, Winzer C, Horl WH, et al. Solid phase detection of C4d-fixing HLA antibodies to predict rejection in high immunological risk kidney transplant recipients. Transpl Int. 2013;26: 121–30. doi:10.1111/tri.12000

25. Lawrence C, Willicombe M, Brookes PA, Santos-Nunez E, Bajaj R, Cook T, et al. Preformed complement-activating low-level donor-specific antibody predicts early antibody-mediated rejection in renal allografts. Transplantation. 2013;95: 341–346. doi:10.1097/TP.0b013e3182743cfa

26. Crespo M, Torio A, Mas V, Redondo D, Pérez-Sáez MJ, Mir M, et al. Clinical relevance of pretransplant anti-HLA donor-specific antibodies: does C1q-fixation matter? Transpl Immunol. 2013;29: 28–33. doi:10.1016/j.trim.2013.07.002

27. Freitas MCS, Rebellato LM, Ozawa M, Nguyen A, Sasaki N, Everly M, et al. The role of immunoglobulin-G subclasses and C1q in de novo HLA-DQ donor-specific antibody kidney transplantation outcomes. Transplantation. 2013;95: 1113–1119. doi:10.1097/TP.0b013e3182888db6

28. Arnold M-L, Ntokou I-S, Doxiadis IIN, Spriewald BM, Boletis JN, Iniotaki AG. Donor-specific HLA antibodies: evaluating the risk for graft loss in renal transplant recipients with isotype switch from complement fixing IgG1/IgG3 to noncomplement fixing IgG2/IgG4 anti-HLA alloantibodies. Transpl Int. 2014;27: 253–61. doi:10.1111/tri.12206

29. Smith JD, Ibrahim MW, Newell H, Danskine AJ, Soresi S, Burke MM, et al. Pre-transplant donor HLA-specific antibodies: characteristics causing detrimental effects on survival after lung transplantation. J Heart Lung Transplant. 2014;33: 1074–1082. doi:10.1016/j.healun.2014.02.033

30. Everly MJ, Rebellato LM, Haisch CE, Briley KP, Bolin P, Kendrick WT, et al. Impact of IgM and IgG3 anti-HLA alloantibodies in primary renal allograft recipients. Transplantation. 2014;97: 494–501. doi:10.1097/01.TP.0000441362.11232.48

31. O’Leary JG, Kaneku H, Banuelos N, Jennings LW, Klintmalm GB, Terasaki PI. Impact of IgG3 subclass and C1q-fixing donor-specific HLA alloantibodies on rejection and survival in liver transplantation. Am J Transplant. 2015;15: 1003–1013. doi:10.1111/ajt.13153

32. Wozniak LJ, Hickey MJ, Venick RS, Vargas JH, Farmer DG, Busuttil RW, et al. Donor-specific HLA Antibodies Are Associated With Late Allograft Dysfunction After Pediatric Liver Transplantation. Transplantation. 2015;99: 1416–22. doi:10.1097/TP.0000000000000796

33. Khovanova N, Daga S, Shaikhina T, Krishnan N, Jones J, Zehnder D, et al. Subclass analysis of donor HLA-specific IgG in antibody-incompatible renal transplantation reveals a significant association of IgG4 with rejection and graft failure. Transpl Int. 2015;28: 1405–1415. doi:10.1111/tri.12648

34. Sicard A, Ducreux S, Rabeyrin M, Couzi L, McGregor B, Badet L, et al. Detection of C3d-binding donor-specific anti-HLA antibodies at diagnosis of humoral rejection predicts renal graft loss. J Am Soc Nephrol. 2015;26: 457–467. doi:10.1681/ASN.2013101144

35. Thammanichanond D, Wiwattanathum P, Mongkolsuk T, Kantachuvesiri S, Worawichawong S, Vallipakorn SA, et al. Role of Pretransplant Complement-fixing Donor-specific Antibodies Identified by C1q Assay in Kidney Transplantation. Transplant Proc. 2016;48: 756–760. doi:10.1016/j.transproceed.2015.12.116

36. Comoli P, Cioni M, Tagliamacco A, Quartuccio G, Innocente A, Fontana I, et al. Acquisition of C3d-Binding Activity by *De Novo* Donor-Specific HLA Antibodies Correlates With Graft Loss in Nonsensitized Pediatric Kidney Recipients. Am J Transplant. 2016;16: 2106–2116. doi:10.1111/ajt.13700

37. Yamamoto T, Watarai Y, Takeda A, Tsujita M, Hiramitsu T, Goto N, et al. De Novo Anti-HLA DSA Characteristics and Subclinical Antibody-Mediated Kidney Allograft Injury. Transplantation. 2016;100: 2194–2202. doi:10.1097/TP.0000000000001012

38. Calp-Inal S, Ajaimy M, Melamed ML, Savchik C, Masiakos P, Colovai A, et al. The prevalence and clinical significance of C1q-binding donor-specific anti-HLA antibodies early and late after kidney transplantation. Kidney Int. 2016;89: 209–216. doi:10.1038/ki.2015.275

39. Malheiro J, Tafulo S, Dias L, Martins LS, Fonseca I, Beirão I, et al. Determining donor-specific antibodies C1q-binding ability improves the prediction of antibody-mediated rejection in HLA-incompatible kidney transplantation. Transpl Int. 2017;30: 347–359. doi:10.1111/tri.12873

40. Visentin J, Chartier A, Massara L, Linares G, Guidicelli G, Blanchard E, et al. Lung intragraft donor-specific antibodies as a risk factor for graft loss. J Heart Lung Transplant. 2016;35: 1418–1426. doi:10.1016/j.healun.2016.06.010

41. Bamoulid J, Roodenburg A, Staeck O, Wu K, Rudolph B, Brakemeier S, et al. Clinical Outcome of Patients with De Novo C1q-Binding Donor-Specific HLA Antibodies after Renal Transplantation: Transplantation. 2017;101: 2165–2174. doi:10.1097/TP.0000000000001487

42. Fichtner A, Süsal C, Höcker B, Rieger S, Waldherr R, Westhoff JH, et al. Association of C1q-fixing DSA with late graft failure in pediatric renal transplant recipients. Pediatr Nephrol. 2016;31: 1157–1166. doi:10.1007/s00467-016-3322-8

43. Guidicelli G, Guerville F, Lepreux S, Wiebe C, Thaunat O, Dubois V, et al. Non-Complement-Binding De Novo Donor-Specific Anti-HLA Antibodies and Kidney Allograft Survival. J Am Soc Nephrol. 2016;27: 615–625. doi:10.1681/ASN.2014040326

44. Wiebe C, Gareau AJ, Pochinco D, Gibson IW, Ho J, Birk PE, et al. Evaluation of C1q Status and Titer of *De Novo* Donor-Specific Antibodies as Predictors of Allograft Survival. Am J Transplant. 2017;17: 703–711. doi:10.1111/ajt.14015

45. Moktefi A, Parisot J, Desvaux D, Canoui-Poitrine F, Brocheriou I, Peltier J, et al. C1q binding is not an independent risk factor for kidney allograft loss after an acute antibody-mediated rejection episode: a retrospective cohort study. Transpl Int. 2017;30: 277–287. doi:10.1111/tri.12905

46. Sicard A, Meas-Yedid V, Rabeyrin M, Koenig A, Ducreux S, Dijoud F, et al. Computer-assisted topological analysis of renal allograft inflammation adds to risk evaluation at diagnosis of humoral rejection. Kidney Int. 2017;92: 214–226. doi:10.1016/j.kint.2017.01.011

47. Das BB, Lacelle C, Zhang S, Gao A, Fixler D. Complement (C1q) Binding De Novo Donor Specific Antibodies and Cardiac-Allograft Vasculopathy in Pediatric Heart Transplant Recipients. Transplantation. 2018;102(3): 502-509.doi:10.1097/TP.0000000000001944

48. Couchonnal E, Rivet C, Ducreux S, Dumortier J, Bosch A, Boillot O, et al. Deleterious impact of C3d-binding donor-specific anti-HLA antibodies after pediatric liver transplantation. Transpl Immunol. 2017;45: 8–14. doi:10.1016/j.trim.2017.08.001

49. Bailly E, Anglicheau D, Blancho G, Gatault P, Vuiblet V, Chatelet V, et al. Prognostic Value of the Persistence of C1q-Binding Anti-HLA Antibodies in Acute Antibody-Mediated Rejection in Kidney Transplantation. Transplantation. 2018;102: 688–698. doi:10.1097/TP.0000000000002002

50. Molina J, Navas A, Agüera M-L, Rodelo-Haad C, Alonso C, Rodríguez-Benot A, et al. Impact of Preformed Donor-Specific Anti-Human Leukocyte Antigen Antibody C1q-Binding Ability on Kidney Allograft Outcome. Front Immunol. 2017;8: 1310. doi:10.3389/fimmu.2017.01310

51. Kauke T, Oberhauser C, Lin V, Coenen M, Fischereder M, Dick A, et al. De novo donorspecific anti-HLA antibodies after kidney transplantation are associated with impaired graft outcome independently of their C1q-binding ability. Transpl Int. 2017;30: 360–370. doi:10.1111/tri.12887
