# Supplementary material for: Complement-activating donor-specific anti-HLA antibodies and solid organ transplant survival: A systematic review and meta-analysis
Source: PLoS Med. 2018 May 25;15(5):e1002572. doi: 10.1371/journal.pmed.1002572 (PMC5969739; doi:10.1371/journal.pmed.1002572)
Supplement: S1 Table — (DOCX) [file pmed.1002572.s006.docx]

**S1 Table: Characteristics of the studies with data available on the primary and secondary endpoints**, (n=37) ordered by the year of publication

| **First author,**  **Journal and year of publication**  (reference) | **Organ,**  **Complement-activating assay**  **(number of patients)** | **Inclusion characteristics Outcome data** | **Population characteristics and comparator used for the outcome** | **Variables in the multivariate models**  **(if present)** |
| --- | --- | --- | --- | --- |
| **Wahrmann et al.**  **Transplant international 2009** [18] | * Kidney  * C4d (+) (21)  * C4d (-) (18) | * Retrospective, single-centre analysis of consecutive adult renal transplants selected based on the presence of pre-transplant DSAs  * Data on allograft survival and rejection | * Clinical indication biopsies  * HR available comparing pre-transplant complement-fixing antibodies with C4d (-) and DSA (-) patients | * Female gender, Recipient age, Donor age, CIT, Donor type (living versus deceased donor), and HLA mismatch |
| **Hönger et al.**  **Transplantation 2010** [19] | * Kidney  * C4d (+) (11)  * C4d (-) (53) | * Retrospective, single-centre analysis of consecutive adult renal transplant recipients with low levels of pre-transplant DSAs  * Data on rejection | * Clinical indication and surveillance biopsies (3 and 6 months) * HR available comparing pre-existing complement-fixing antibodies with C4d (-) patients |  |
| **Sutherland et al.** **Pediatric Transplantation 2011** [20] | * Kidney  * C1q (+) (15)  * C1q (-) (20) | * Retrospective, single-centre analysis of paediatric patients without DSAs at the time of transplantation  * Data on allograft survival | * Clinical indication and surveillance biopsies (3,6,12,24 months)  * HR available comparing *de novo* DSA complement-fixing antibodies with C1q (-) patients |  |
| **Hönger et al.**  **Transplantation 2011** [21] | * Kidney  * IgG3 (+) (21)  * IgG3 (-) (4) | * Retrospective, single-centre analysis of adult renal transplant recipients with high levels of DSAs  pre-transplant recipients who developed AMR within 6 months * Data on rejection | * Clinical indication and surveillance biopsies (3 and 6 months) * HR available comparing pre-existing complement-fixing antibodies with a mixture of strong and weak/no complement-activating subclasses |  |
| **Smith et al.**  **American Journal of transplantation 2011** [22] | * Heart  * C4d (+) (26)  * C4d (-) (22) | * Retrospective, single-centre analysis of living heart transplant patients after one year of transplantation without DSAs pre-transplant  * Data on allograft survival | * Clinical indication and surveillance biopsies  * Acute rejection was measured as BPAR in the first year and treated rejection episodes. Treated rejection refers to patients who were treated for rejection for clinical reasons, not always associated with a positive biopsy.  * HR available comparing *de novo* complement-fixing antibodies with DSA (-) patients | * *De novo*-persistent DSAs, HLA-DR mismatch, Haemodynamic compromise, Donor age |
| **Kaneku et al.**  **Liver transplantation 2012** [23] | * Liver  * IgG3 (+) (12)  * IgG3 (-) (24) | * Retrospective (2-centre) analysis of adult liver transplant recipients with liver biopsies showing chronic rejection and DSA analysis at the same time  * Data on allograft survival | * Clinical indication biopsies  * Biopsy-proven chronic ductopenic rejection  * HR available comparing pre-existing and *de novo* DSA complement-fixing antibodies with IgG without IgG3 subclasses | * IgG3 DSAs; Multiple IgG subclass DSAs; Donor age: <50 versus >50 years; Donor age (years) Recipient race; Recipient age; Cytomegalovirus ever; ACR during the first year; SRR during the first year; ACR ever; SRR ever; Cyclosporine versus tacrolimus at 1 month |
| **Bartel et al. Transplant International 2013** [24] | * Kidney * C4d (+) (44)  * C4d (-) (24) | * Retrospective, single-centre analysis of 68 desensitized patients who had been subjected to peri-transplant desensitization  * Data on rejection | * Clinical indication biopsies  * HR available comparing pre-existing DSA complement-fixing antibodies with C4d (-) patients | * Female gender, Number of transplants, History of pregnancy, Number of HLA mismatches, Positive CDCXM, and Number of IgG DSA |
| **Lawrence et al. Transplantation 2013** [25] | * Kidney  * C4d (+) (10)  * C4d (-) (42) | * Retrospective, single-centre study of consecutive kidney transplant patients  * Data on rejection | * Clinical indication biopsies  * HR available comparing pre-transplant complement-fixing antibodies with C4d (-) patients | * Age at transplant, Number of transplants, Number of mismatches, Number of DSA, Class DSA, MFI iDSA, Sum MFI, Complement-activating DSA |
| **Crespo et al.**  **Transplant Immunology 2013** [26] | * Kidney  * C1q (15)  * C1q (-) (13) | * Retrospective (2-centre) analysis of patients with pre-transplant DSAs  * Data on allograft survival and rejection | * Clinical indication biopsies  * HR available comparing pre-transplant complement-fixing antibodies with C1q (-) patients |  |
| **Loupy et al.** **New England Journal of Medicine 2013** [9] | * Kidney  * C1q (+) (77)  * C1q (-) (239) | * Consecutive adult patients in a retrospective (2-centre) analysis. Unselected global population with DSA detection before or after transplantation  * Data on allograft survival | * Clinical indication and surveillance biopsies (3-12 months)  * HR available comparing complement pre-existing and *de novo* DSA-fixing antibodies with a group of C1q (-) and DSA (-) patients | * Age donor, CIT, Deceased donor, Female donor, Subsequent transplant, GFR, Histologic factors, DSA, C1q (+) DSA |
| **Freitas et al.**  **Transplantation 2013** [27] | * Kidney  * IgG3 (+) (24)  * IgG3 (-) (30) | * Retrospective, single-centre analysis of patients selected on the basis of DSA detection during follow-up  * Data on allograft survival | * Clinical indication biopsies  * HR available comparing *de novo* complement-fixing antibodies with IgG2/IgG4 group and DSA (-) patients and C1q (+) vs C1q (-) patients | * Recipient ethnicity, HLA-A/B/DR/DQ mismatch, Type of allograft, Cause of end-stage renal disease, Occurrence of delayed graft function, and Study group |
| **Arnold et al.**  **Transplant International 2014** [28] | * Kidney  * IgG3 (+) (74)  * IgG3 (-) (20) | * Retrospective, single-centre analysis of patients without DSAs pre-transplant and screened for *de novo* DSAs  * Data on allograft survival | * Clinical indication biopsies  * HR available comparing *de novo* complement-fixing antibodies with DSA (-) patients |  |
| **Smith et al.**  **Journal of Heart and Lung transplantation 2014** [29] | * Lung  * C4d (+) (9)  * C4d (-) (18) | * Retrospective, single-centre analysis of patients with pre-transplant DSA detection  * Data on allograft survival | * No biopsy performed  * HR available comparing pre-transplant complement-fixing antibodies with a group of C4d (-) and DSA (-) patients | * DSA, C4d DSA, Indication for transplant, Type of operation, Recipient age, HLA-A mismatch, Recipient FEV |
| **Everly et al.** **Transplantation 2014** [30] | * Kidney  * IgG3 (+) (25)  * IgG3 (-) (22) | * Retrospective, single-centre analysis of primary kidney transplant patients without pre-transplant DSA detection  * Data on allograft survival | * Clinical indication biopsies  * HR available comparing *de novo* complement-fixing antibodies with a group of IgG3 (-) and DSA (-) patients |  |
| **O’Leary et al.**  **American Journal of Transplantation 2015** [31] | * Liver  * IgG3 (+) (94) / C1q (+) (53)  * IgG3 (-) (90) / C1q (-) (131) | * Retrospective, single-centre analysis of consecutive patients with 1-year survival post-transplantation  * One group analysed pre-transplant DSA effects and another group analysed the impact of *de novo* DSAs  * Data on allograft survival | * No biopsy performed  * HR available comparing either pre-transplant or *de novo* complement-fixing antibodies with DSA (-) patients | * DSA, IgG3 (+), and C1q (+) DSA and variables input into the model: Cytomegalovirus infection, low CNI levels, HCV RNA positivity, recipient age, and donor age |
| **Wozniak et al. Transplantation 2015** [32] | * Liver  * C1q (+) (13)  * C1q (-) (14) | * Retrospective, single-centre analysis of paediatric liver transplant patients who were either non-tolerant, tolerant or stable  * Data on rejection | * Biopsy-proven chronic rejection or acute cellular rejection  * HR available comparing pre-transplant and *de novo* complement-fixing antibodies with a group of C1q (-) and DSA (-) patients | * Primary diagnosis of cholestatic liver disease, Male sex, Race, Recipient age at transplant, Time since transplant, Re-transplant (recipient), History of early rejection, History of PTLD, Nontolerant clinical phenotype, DSA, and C1q-binding DSA |
| **Khovanova et al.** **Transplant International 2015** [33] | * Kidney  * IgG3 (+) preformed and *de novo* DSA (27) / Preformed DSA (35)  * IgG3 (-) preformed and *de novo* DSA (53) / Preformed DSA (45) | * Retrospective, single-centre analysis of HLA-incompatible desensitized patients  * Data on allograft survival | * Clinical indication biopsies  * HR available comparing pre-existing and *de novo* complement-fixing antibodies with other IgG subclasses | * Previous transplant, CDCXM (+), Single highest IgG DSA, IgG3, IgG1, IgG2, IgG4, DGF) |
| **Sicard et al.** **Journal of the American Society of Nephrology 2015** [34] | * Kidney  * C3d (+) (40) / C1q (+) (30)  * C3d (-) (29) / C1q (-) (35) | * Retrospective analysis of consecutive (2-centre) adult patients who developed ABMR  * Data on allograft survival | * Clinical indication and surveillance biopsies (3-12 months)  * HR available comparing pre-existing and *de novo* complement-fixing antibodies with C3d and C1q (-) | * GFR, Proteinuria, IFTA, C3d binding DSA, MFI |
| **Thammanichanond et al.**  **Transplantation Proceedings 2016** [35] | * Kidney  * C1q (+) (12)  * C1q (-) (36) | * Retrospective, single-centre cohort study of patients with pre-transplant DSAs  * Data on rejection | * Clinical indication biopsies  * HR available comparing pre-existing and *de novo* complement-fixing antibodies with C1q (-) patients |  |
| **Comoli et al.**  **American Journal of Transplantation 2016** [36] | * Kidney * C3d (+) (9) / C1q (+) (25)  * C3d (-) (105) / C1q (-) (89) | * Retrospective analysis of consecutive paediatric recipients, single-centre, first kidney transplant without any HLA antibodies in sera or at the time of transplantation  * Data on allograft survival and rejection | * Clinical indication biopsies  * HR available comparing *de novo* complement-fixing antibodies with C3d (-) or C1q (-) patients |  |
| **Yamamoto et al.**  **Transplantation 2016** [37] | * Kidney  * C1q (+) (21)  * C1q (-) (22) | * Retrospective analysis of patients with *de novo* DSAs and surveillance biopsies  * Data on rejection | * Surveillance biopsies  * HR available comparing *de novo* complement-fixing antibodies with C1q (-) patients |  |
| **Calp-Inal et al.**  **Kidney International 2016** [38] | * Kidney  * C1q (+) (11 (group 1)  * C1q (-) (20 (group 1) | * Retrospective analysis, single-centre, consecutive transplant patients:  - Group 1 without pre-transplant DSAs  - Group 2 with a mix of pre-existing and *de novo* DSAs  * Data on allograft survival | * Clinical indication biopsies  * HR available comparing pre-existing and *de novo* complement-fixing antibodies with DSA (-) patients |  |
| **Malheiro et al.**  **Transplant International 2016** [39] | * Kidney  * C1q (+) (13)  * C1q (-) (47) | * Retrospective, single-centre analysis of kidney transplant patients with DSAs pre-transplant  * Data on rejection | * Clinical indication biopsies  * HR available comparing preformed complement-fixing antibodies with C1q (-) patients | * Peak PRA, DSA presence, HLA mismatch, MFI >1500 and C1q (+) |
| **Visentin et al.** **Journal of lung and heart transplantation 2016** [40] | * Lung  * C1q (+) (12)  * C1q (-) (16) | * Retrospective, single-centre analysis of patients with biopsy (with demonstration of rejection) and serum available  * Data on allograft survival | * Clinical indication and surveillance biopsies  * Biopsy-proven rejection from ISHLT 2013  * HR available comparing pre-existing and *de novo* complement-fixing antibodies with a group of C1q (-) and DSA (-) patients | * Infection, Severe BOS, sDSA, gDSA |
| **Kauke et al.** **Transplantation 2016** [27] | * Kidney  * C1q (+) (27)  * C1q (-) (13) | * Retrospective, single-centre analysis of patients selected based on biopsy-proven rejection during graft dysfunction or BK viremia  * Data on allograft survival and rejection | * Clinical indication biopsies  * HR available comparing *de novo* complement-fixing antibodies with DSA (-) patients | * DGF, re-transplantation, acute rejection episodes, renal function at 1, 2, and 3 years post-transplant, *de novo* non-DSA C1q (+), de *novo* DSA C1q (-), and *de novo* DSA C1q (+)) |
| **Bamoulid et al.**  **Transplantation 2016** [41] | * Kidney  * C1q (+) (36)  * C1q (-) (23) | * Retrospective, single-centre analysis of consecutive patients without DSAs pre-transplant  * Data on allograft survival and rejection | * Clinical indication biopsies  * HR available comparing *de novo* complement-fixing antibodies with C1q (-) patient | * DGF, TCMR prior to ABMR, C1q (+), DSA class I (+), plasmapheresis, lowest post-transplant creatinine and donor age |
| **Fichtner et al.**  **Pediatric Nephrology 2016** [42] | * Kidney  * C1q (+) (9)  * C1q (-) (17) | * Retrospective, single-centre analysis of prospectively screened paediatric patients, non-pre-sensitized  * Data on allograft survival | * Clinical indication biopsies later than 1-year post-transplant  * HR available comparing pre-existing and *de novo* complement-fixing antibodies with DSA (-) patients | * eGFR at the time of index biopsy, presence of transplant glomerulopathy, C4d-positivity in index biopsy, and C1q DSA-positivity |
| **Guidicelli et al.**  **Journal of the American Society of Nephrology 2016** [43] | * Kidney  * C1q (+) (12)  * C1q (-) (13) | * Retrospective, single-centre analysis of consecutive non-sensitized patients  * Data on allograft survival | * Clinical indication biopsies  * HR available comparing *de novo* complement-fixing antibodies with a group of C1q (-) and DSA (-) patients |  |
| **Lefaucheur et al.  Journal of the American Society of Nephrology 2016** [10] | * Kidney  * IgG3 (+) (35) / C1q (+) (52)  * IgG3 (-) (90) / C1q (-) (73) | * Retrospective analysis of consecutive patients (2-centre), patients were unselected  * Data on allograft survival | * Clinical indication and surveillance biopsies (3-12 months)  * HR available comparing pre-existing and *de novo* complement-fixing antibodies with IgG3 (-) or C1q (-) patients | * iDSA HLA class specificity, iDSA MFI, iDSA C1q-binding capacity, and iDSA IgG1–4 subclasses |
| **Viglietti et al.** **Journal of the American Society of Nephrology 2017** [11] | * Kidney  * IgG3 (+) (42) / C1q (+) (57)  * IgG3 (-) (144) / C1q (-) (129) | * Retrospective analysis of consecutive patients (2-centre), patients were unselected  * Data on allograft survival | * Clinical indication and surveillance biopsies (12-24 months)  * HR available comparing pre-existing and *de novo* complement-fixing antibodies with a group of patients with IgG3 (-) or C1q (-) and DSA (-) | * DSA HLA class, iDSA MFI level, iDSA C1q binding capacity, and iDSA IgG1–4 subclasses |
| **Wiebe et al.**  **American Journal of Transplantation 2017** [44] | * Kidney  * C1q (+) (17)  * C1q (-) (53) | * Retrospective analysis of consecutive adults and paediatrics patients, single-centre. Patients without pre-transplant sensitization  * Data on allograft survival | * Surveillance biopsies  * HR available comparing *de novo* complement-fixing antibodies with C1q (-) patients | * C1q (+), Non-adherence, Clinical versus subclinical phenotypes, *de novo* DSA titer >1:64, *de novo* DSA titer >1:1024) |
| **Moktefi et al.**  **Transplant International 2017** [45] | * Kidney  * C1q (+) (20)  * C1q (-) (25) | * Retrospective analysis (2-centre) of patients selected based on the development of acute ABMR and the presence of DSA  * Data on allograft survival | * Clinical indication biopsies  * HR available comparing pre-existing and *de novo* complement-fixing antibodies with C1q (-) patients |  |
| **Sicard et al.**  **Kidney international 2017** [46] | * Kidney  * C3d (+) (21)  * C3d (-) (31) | * Retrospective analysis of consecutive adult kidney transplant patients (2-centre) with unselected patients  * Data on allograft survival | * Clinical indication and surveillance biopsies (3-12 months)  * HR available comparing pre-existing and *de novo* complement-fixing antibodies with C3d (-) patients | * eGFR, proteinuria, DSA MFI, DSA-C3d status, number of macrophages in interstitium) |
| **Bibhuti et al. Transplantation 2017** [47] | * Heart  * C1q (+) (37)  * C1q (-) (22) | * Retrospective, single-centre analysis of paediatrics heart transplant without DSA pre-transplantation and at the time of transplantation * Data on allograft survival | * Clinical indication and surveillance biopsies based on their own ABMR diagnosis protocol  * HR available comparing *de novo* complement-fixing antibodies with C1q (-) patients | * C1q (+) DSA, Pretransplant renal insufficiency, Pretransplant ventilator use |
| **Couchonnal et al.**  **Transplant Immunology 2017** [48] | * Liver  * C3d (+) (18)  * C3d (-) (6) | * Retrospective analysis, single-centre analysis of consecutive paediatrics liver transplant selected on the presence of DSA during follow-up  * Data on allograft survival | * Biopsy-proven rejection from the 2000 Banff  * Clinical indication and surveillance biopsy (1, 5, 10, 15, and 20 year)  * HR available compared pre-existing and *de novo* complement fixing antibodies with C3d (-) patients |  |
| **Bailly et al.**  **Transplantation 2017** [49] | * Kidney  * C1q (+) (17)  * C1q (-) (8) | * Retrospective analysis of multicentre, prospective, randomized, double-blind, placebo-controlled, parallel-group trials. Patients were selected on the basis of ABMR development and DSA detection. Patients treated either with standard of care (PP+IgIV) or Rituximab + standard of care  * Data on allograft survival | * Clinical indication and surveillance biopsies  * HR available comparing pre-existing and *de novo* complement-fixing antibodies with C1q (-) patients |  |
| **Molina et al.**  **Frontiers in Immunology** [50] | * Kidney  * C1q (+) (30)  * C1q (-) (62) | * Retrospective analysis, single-centre analysis of consecutive adult kidney transplant patients selected on pre-transplant DSA detection  * Data on allograft survival | * No information on biopsies  * HR available comparing pre-transplant complement-fixing antibodies with DSA (-) patients | * C1q (+) DSA, Donor age, CIT, HLA-DR mismatch |

ABMR: Antibody-Mediated Rejection; ACR: Acute Cellular Rejection; BOS: Bronchiolitis Obliterans Syndrome; BPAR: Biopsy Proven Acute Rejection; CIT: Cold Ischaemia Time; CDCXM: Complement-Dependent Cytotoxicity Cross-Match; CNI: Calcineurin Inhibitor; DSA: Donor-Specific Antibody; DGF: Delayed Graft Function; eGFR: estimated Glomerular Filtration Rate; FEV: Forced Expiratory Volume; gDSA: Graft DSA; HCV: Hepatitis C Virus; HLA: Human Leucocyte Antigen; ISHLT: International Society of Heart and Lung Transplantation; iDSA: Immuno-Dominant DSA; IA: Immunoadsorption; IgIV: Intra-venous Immunoglobulin; IFTA: Interstitial Fibrosis and Tubular Atrophy; MFI: Mean Fluorescence Intensity; PRA: Panel Reactive Antigen; PP: Plasmapheresis; sDSA: Serum DSA; SRR: Steroids Resistant Rejection; TCMR: T-Cell Mediated Rejection; TG: Transplant Glomerulopathy; Tx: Transplantation.
