# Supplementary material for: Complement-activating donor-specific anti-HLA antibodies and solid organ transplant survival: A systematic review and meta-analysis
Source: PLoS Med. 2018 May 25;15(5):e1002572. doi: 10.1371/journal.pmed.1002572 (PMC5969739; doi:10.1371/journal.pmed.1002572)
Supplement: S2 Table — (DOCX) [file pmed.1002572.s007.docx]

**S2 Table: Methodological quality assessment of non-randomized trials** New Castle-Ottawa Scale evaluation of the observational studies included in the meta-analysis (n=36), stratified by organ type.

| **Authors (years) (reference)** | **Type of study** | **Type of C’ anti-HLA DSA (Organ)** | **NOS Classification** | | |
| --- | --- | --- | --- | --- | --- |
|  |  |  | **Selection** | **Comparability** | **Outcome** |
| Sutherland et al. (2011) [20] | Cohort | C1q (Kidney) | *** | ** | ** |
| Freitas et al. (2013) [27] | Cohort | IgG3/C1q (Kidney) | **** | * | ** |
| Loupy et al. (2013) [9] | Cohort | C1q (Kidney) | **** | ** | ** |
| Crespo et al. (2013) [26] | Cohort | C1q (Kidney) | **** |  | * |
| Thammanichanond et al. (2016) [35] | Cohort | C1q (Kidney) | *** |  | * |
| Fichtner et al. (2016) [42] | Cohort | C1q (Kidney) | **** | * | *** |
| Calp-Inal et al. (2016) [38] | Cohort | C1q (Kidney) | *** | * | * |
| Guidicelli et al. (2016) [43] | Cohort | C1q (Kidney) | **** | * | *** |
| Bamoulid et al. (2016) [41] | Cohort | C1q (Kidney) | *** |  | *** |
| Kauke et al. (2016) [51] | Cohort | C1q (Kidney) | **** | * | ** |
| Yamamoto et al. (2016) [37] | Cohort | C1q (Kidney) | *** | * | ** |
| Moktefi A. et al. (2017) [45] | Cohort | C1q (Kidney) | *** | * | * |
| Wiebe et al. (2017) [44] | Cohort | C1q (Kidney) | *** | * | ** |
| Malheiro et al. (2017) [39] | Cohort | C1q (Kidney) | **** | * | * |
| Sicard et al. (2015) [34] | Cohort | C3d/C1q (Kidney) | **** | * | * |
| Comoli et al. (2016) [36] | Cohort | C3d/C1q (Kidney) | *** |  | * |
| Molina et al. (2017) [50] | Cohort | C1q (Kidney) | *** | * | * |
| Sicard et al. (2017) [46] | Cohort | C3d (Kidney) | *** | ** | ** |
| Wahrmann et al. (2009) [18] | Cohort | C4d (Kidney) | *** | * | ** |
| Hönger et al. (2010) [19] | Cohort | C4d (Kidney) | *** | * | * |
| Bartel et al. (2013) [24] | Cohort | C4d (Kidney) | *** | * | ** |
| Lawrence et al. (2013) [25] | Cohort | C4d (Kidney) | *** | * | * |
| Hönger et al. (2011) [21] | Cohort | IgG3 (Kidney) | *** | * | * |
| Arnold et al. (2014) [28] | Cohort | IgG3 (Kidney) | **** |  | * |
| Everly et al. (2014) [30] | Cohort | IgG3 (Kidney) | *** | * | ** |
| Khovanova et al. (2015) [33] | Cohort | IgG3 (Kidney) | *** | * | ** |
| Lefaucheur et al. (2016) [10] | Cohort | IgG3/C1q (Kidney) | **** | ** | * |
| Viglietti et al. (2017) [11] | Cohort | IgG3/C1q (Kidney) | **** | ** | *** |
| Smith J. et al. (2011) [22] | Cohort | C4d (Heart) | **** | * | * |
| Bibhuti B. et al. (2017) [47] | Cohort | C1q (Heart) | **** | ** | * |
| Kaneku H. et al. (2012) [23] | Case control | IgG3 (Liver) | *** | ** | * |
| O’Leary J. et al. (2015) [31] | Cohort | IgG3/C1q (Liver) | **** | * | * |
| Wozniak L. et al. (2015) [32] | Cohort | C1q (Liver) | * |  | ** |
| Couchonnal et al. (2017) [48] | Cohort | C3d (Liver) | **** | ** | * |
| Smith J. et al. (2014) [29] | Cohort | C4d (Lung) | **** | * | * |
| Visentin J. et al. (2016) [40] | Cohort | C1q (Lung) | **** | * | * |

The study by Bailly et al. (2017) was assessed as a randomized control trial using the Cochrane Risk of bias tool.

C’ anti-HLA DSA: complement-activating anti-human leucocyte antigen donor-specific antibodies.
