# Supplementary figures and images for: Complement-activating donor-specific anti-HLA antibodies and solid organ transplant survival: A systematic review and meta-analysis
Source: PLoS Med. 2018 May 25;15(5):e1002572. doi: 10.1371/journal.pmed.1002572 (PMC5969739; doi:10.1371/journal.pmed.1002572)

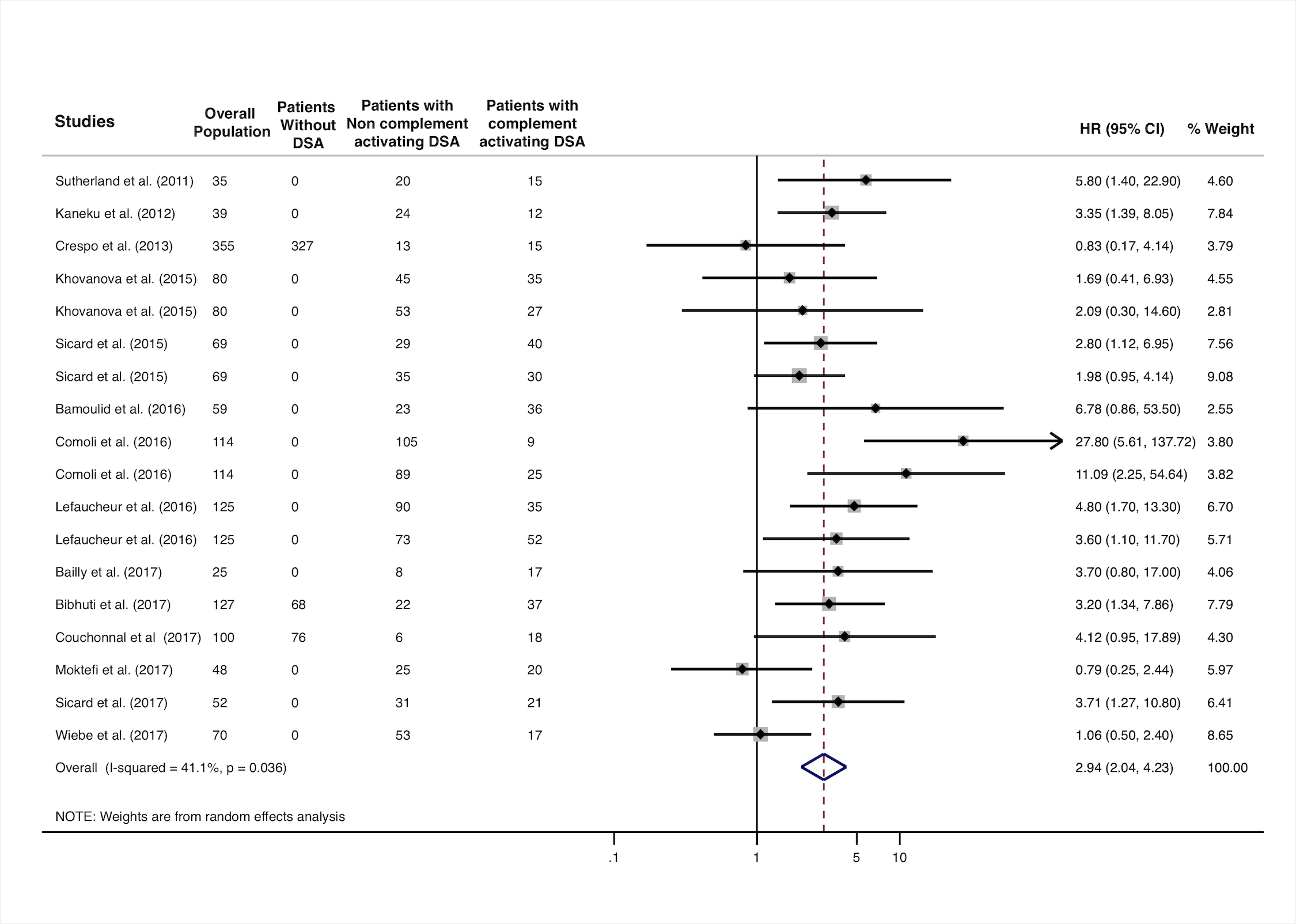

Supplement: S1 Fig — Studies comparing complement-activating anti-HLA DSAs with non–complement-activating anti-HLA DSAs. Studies are listed by the date of publication. The black diamond-shaped boxes represent the HR for each individual study. The grey boxes around the black diamond represent the weight of the study, and lines represent the 95% CI for individual studies. The blue diamond at the end represents the overall HR. The number of patients in the overall population does not correspond to the sum of the different groups for the studies of Kaneku et al. (2012) (3 patients), Sicard et al. (2015) (4 patients), and Moktefi et al. (2017) (3 patients) either because the data for these patients were missing or because they were not involved in the analysis. CI, confidence interval; DSA, donor-specific antibody; HLA, human leukocyte antigen; HR, hazard ratio. (TIFF) [file pmed.1002572.s008.tiff]

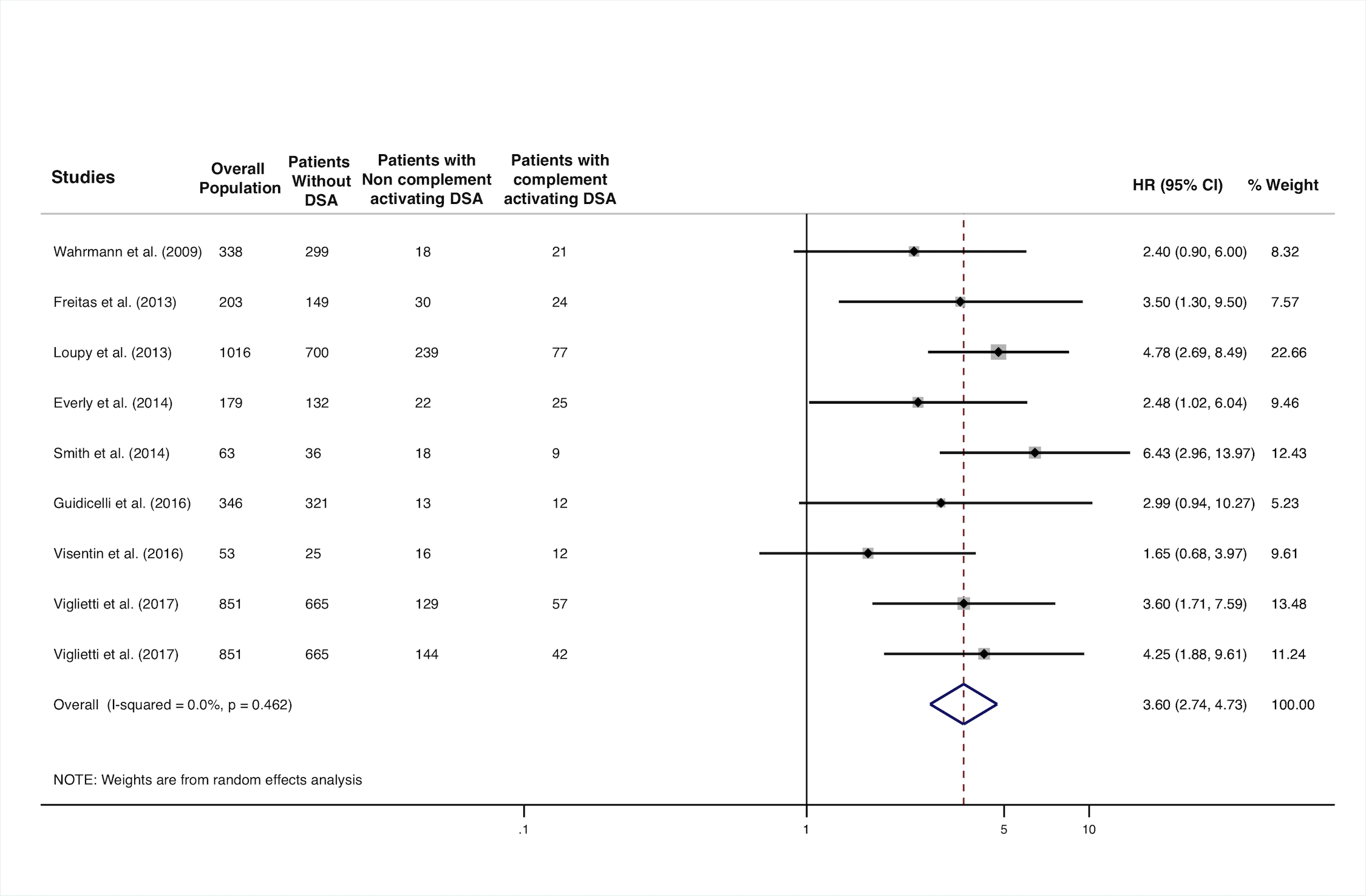

Supplement: S2 Fig — Studies comparing complement-activating anti-HLA DSAs with a mixed group of patients without anti-HLA DSAs and with non–complement-activating anti-HLA DSAs. Studies are listed by the date of publication. The black diamond-shaped boxes represent the HR for each individual study. The grey boxes around the black diamond represent the weight of the study, and lines represent the 95% CI for individual studies. The blue diamond at the end represents the overall HR. CI, confidence interval; DSA, donor-specific antibody; HLA, human leukocyte antigen; HR, hazard ratio. (TIFF) [file pmed.1002572.s009.tiff]

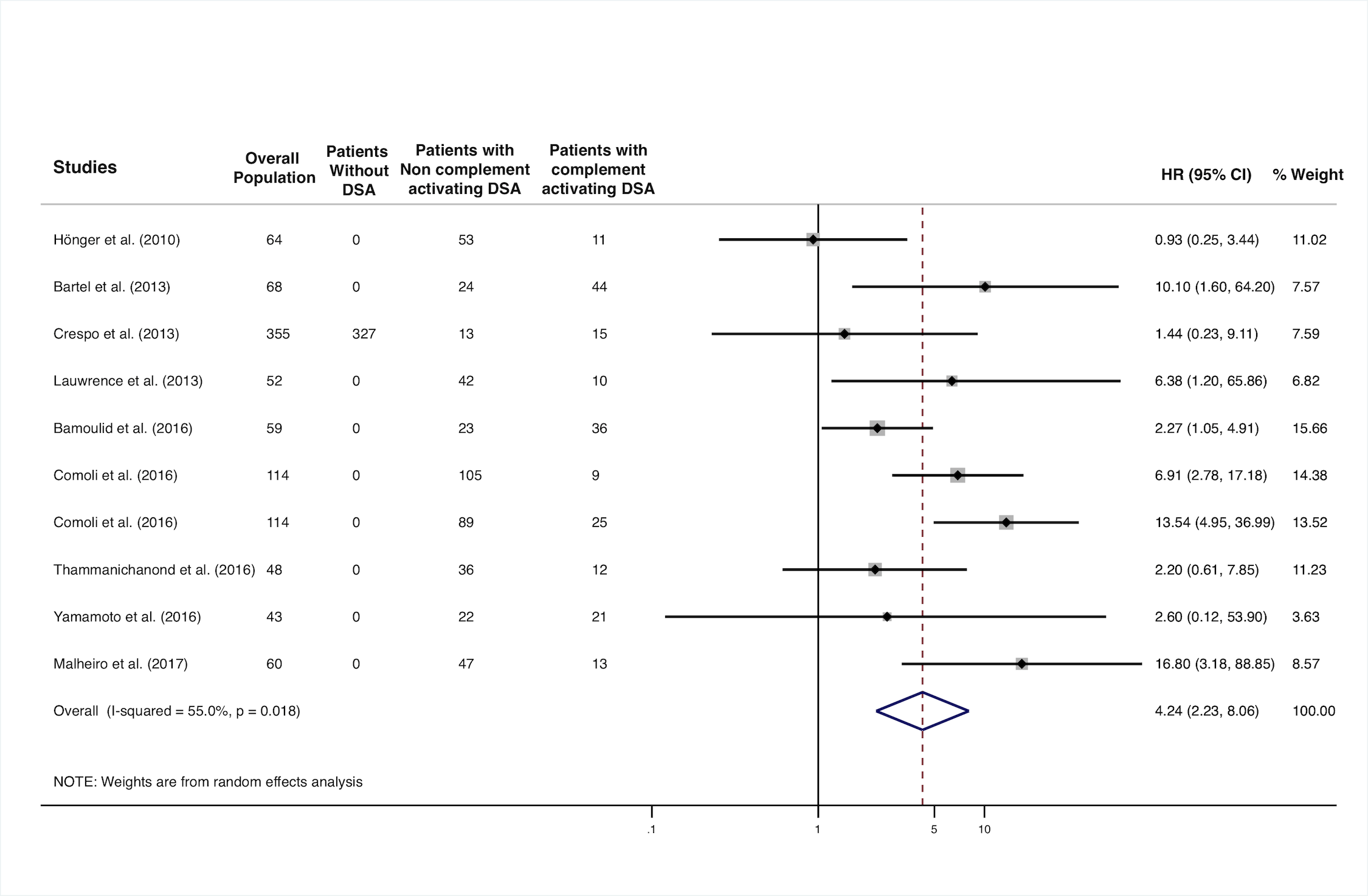

Supplement: S3 Fig — Studies comparing complement-activating anti-HLA DSAs with non–complement-activating anti-HLA DSAs. Studies are listed by date of publication. The black diamond-shaped boxes represent the HR for each individual study. The grey boxes around the black diamond represent the weight of the study, and lines represent the 95% CI for individual studies. The blue diamond at the end represents the overall HR. CI, confidence interval; DSA, donor-specific antibody; HLA, human leukocyte antigen; HR, hazard ratio. (TIFF) [file pmed.1002572.s010.tiff]

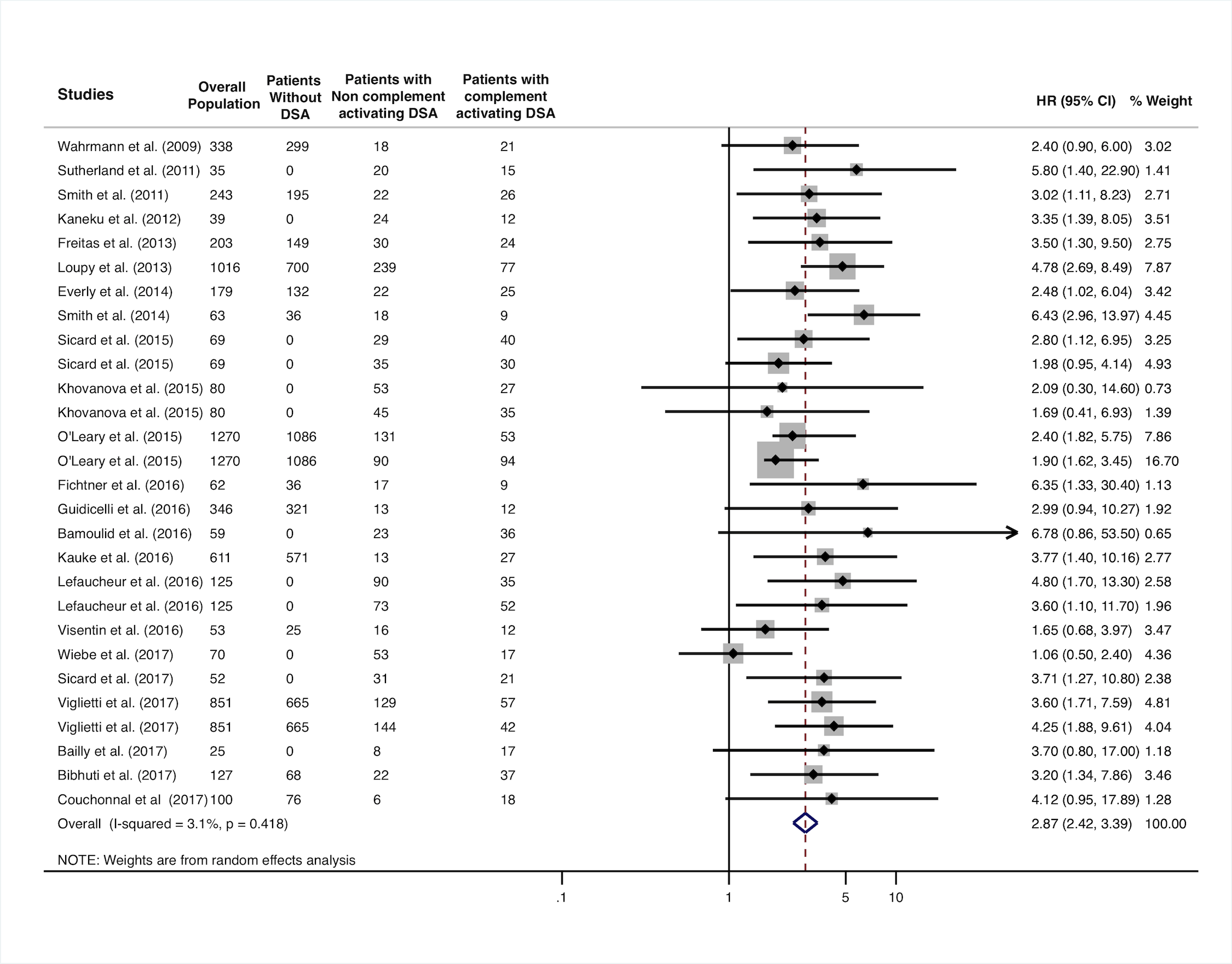

Supplement: S4 Fig — Studies are listed by date of publication. The black diamond-shaped boxes represent the HR for each individual study. The grey boxes around the black diamond represent the weight of the study, and lines represent the 95% CI for individual studies. The blue diamond at the end represents the overall HR. The number of patients in the overall population does not correspond to the sum of the different groups for the studies of Kaneku et al. (2012) (3 patients) and Sicard et al. (2015) (4 patients) either because the data for these patients were missing or because they were not involved in the analysis. CI, confidence interval; DSA, donor-specific antibody; HLA, human leukocyte antigen; HR, hazard ratio. (TIFF) [file pmed.1002572.s011.tiff]

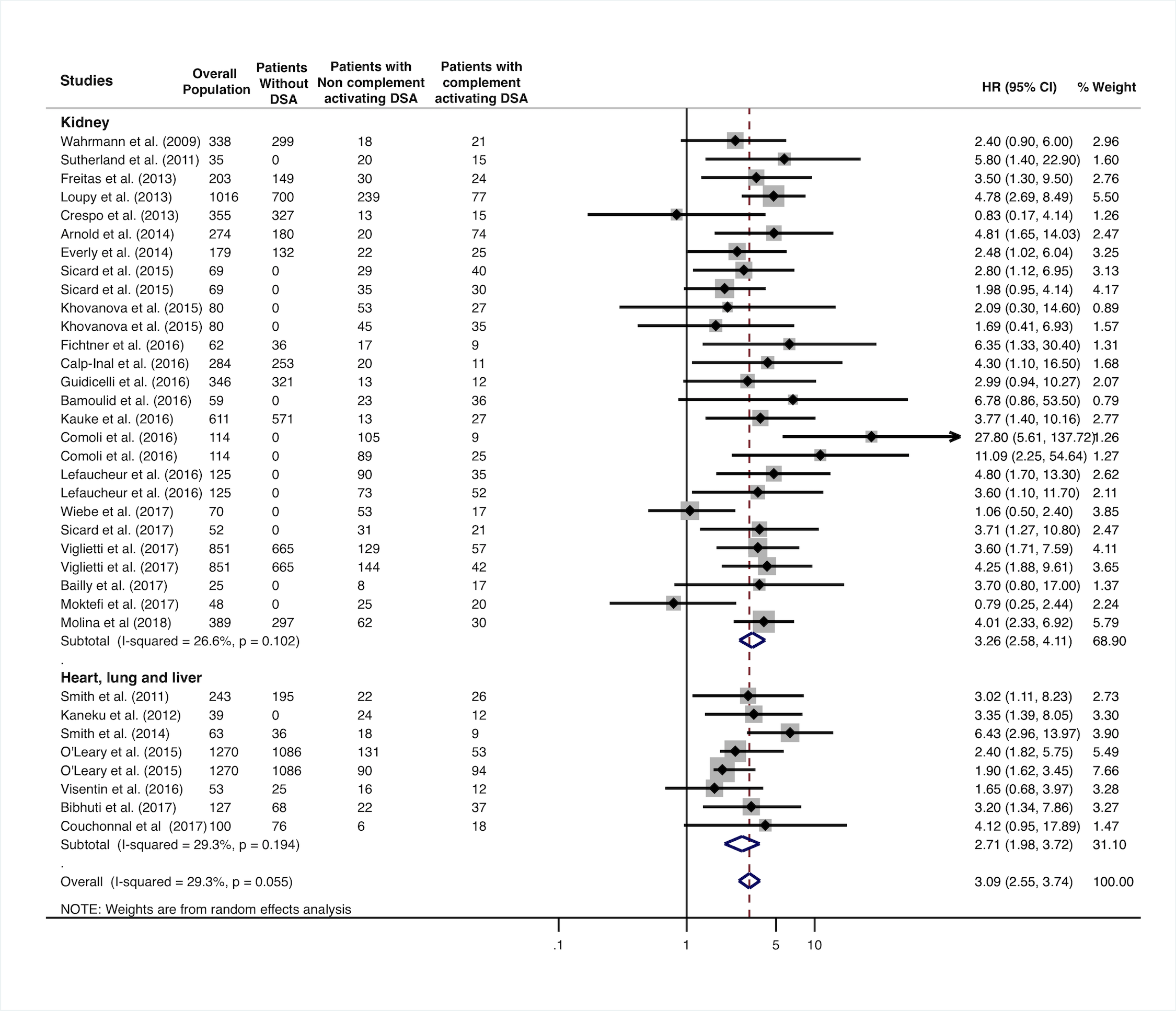

Supplement: S5 Fig — Studies are listed by date of publication. The black diamond-shaped boxes represent the HR for each individual study. The grey boxes around the black diamond represent the weight of the study, and lines represent the 95% CI for individual studies. The blue diamond at the end represents the overall HR. Number of patients in the overall population does not correspond to the sum of the different groups for the studies of Kaneku et al. (2012) (3 patients), Sicard et al. (2015) (4 patients), and Moktefi et al. (2017) (3 patients) either because the data for these patients were missing or because they were not involved in the analysis. CI, confidence interval; DSA, donor-specific antibody; HLA, human leukocyte antigen; HR, hazard ratio. (TIFF) [file pmed.1002572.s012.tiff]

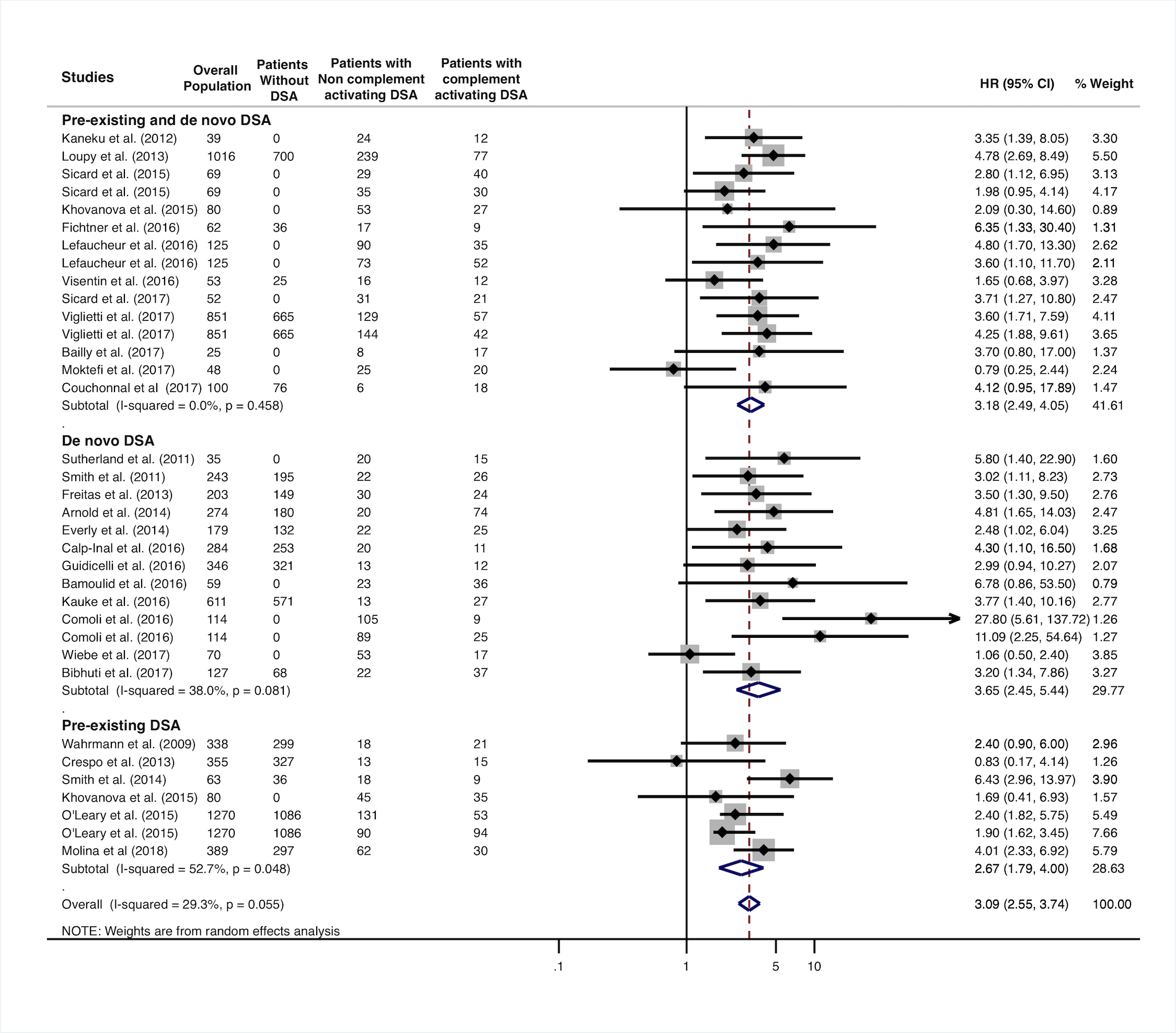

Supplement: S6 Fig — Studies are listed by date of publication. The black diamond-shaped boxes represent the HR for each individual study. The grey boxes around the black diamond represent the weight of the study, and lines represent the 95% CI for individual studies. The blue diamond at the end represents the overall HR. Number of patients in the overall population does not correspond to the sum of the different groups for the studies of Kaneku et al. (2012) (3 patients), Sicard et al. (2015) (4 patients), and Moktefi et al. (2017) (3 patients) either because the data for these patients were missing or because they were not involved in the analysis. CI, confidence interval; DSA, donor-specific antibodies; HLA, human leukocyte antigen; HR, hazard ratio. (TIFF) [file pmed.1002572.s013.tiff]

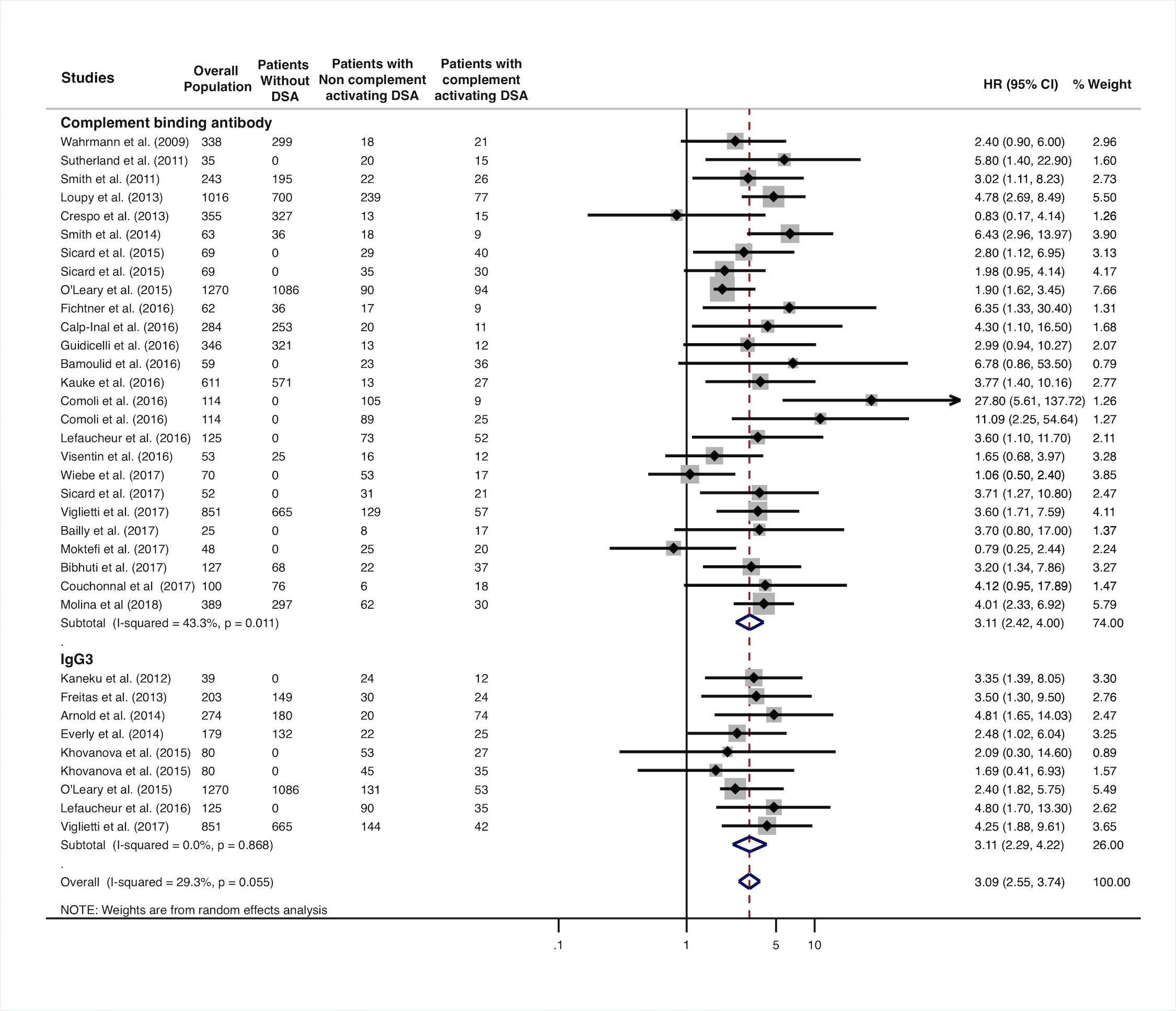

Supplement: S7 Fig — Studies are listed by date of publication. The black diamond-shaped boxes represent the HR for each individual study. The grey boxes around the black diamond represent the weight of the study, and lines represent the 95% CI for individual studies. The blue diamond at the end represents the overall HR. The number of patients in the overall population does not correspond to the sum of the different groups for the studies of Kaneku et al. (2012) (3 patients), Sicard et al. (2015) (4 patients), and Moktefi et al. (2017) (3 patients) either because the data for these patients were missing or because they were not involved in the analysis. CI, confidence interval; DSA, donor-specific antibody; HLA, human leukocyte antigen; HR, hazard ratio. (TIFF) [file pmed.1002572.s014.tiff]

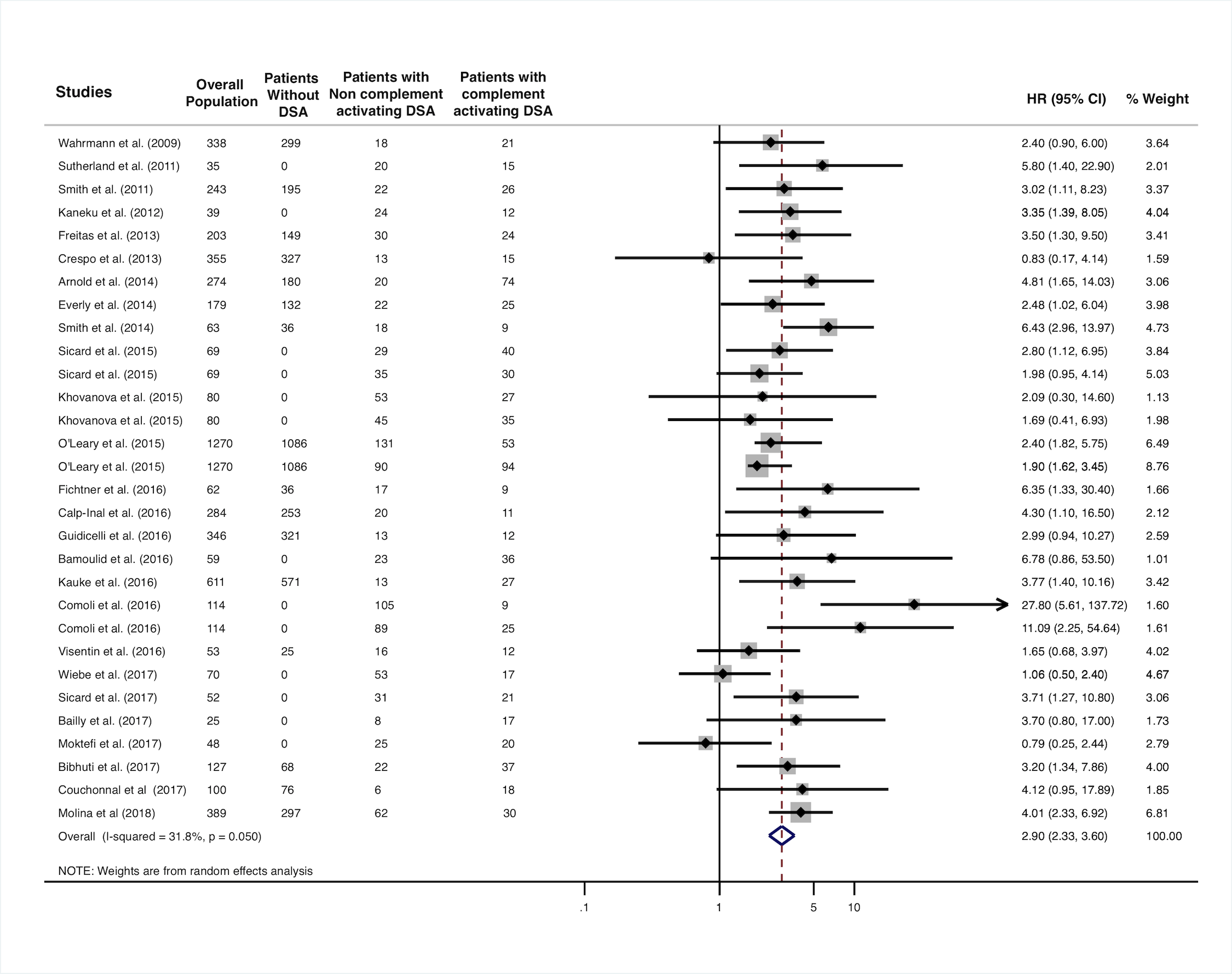

Supplement: S8 Fig — Studies are listed by the date of publication. The black diamond-shaped boxes represent the HR for each individual study. The grey boxes around the black diamond represent the weight of the study, and lines represent the 95% CI for individual studies. The blue diamond at the end represents the overall HR. The number of patients in the overall population does not correspond to the sum of the different groups for the studies of Kaneku et al. (2012) (3 patients), Sicard et al. (2015) (4 patients), and Moktefi et al. (2017) (3 patients) either because the data for these patients were missing or because they were not involved in the analysis. CI, confidence interval; DSA, donor-specific antibody; HLA, human leukocyte antigen; HR, hazard ratio. (TIFF) [file pmed.1002572.s015.tiff]
